# Bispecific antibodies in the treatment of epithelial ovarian, endometrial, and cervical cancer: an overview of current applications, challenges, and emerging opportunities

**DOI:** 10.37349/etat.2025.1002345

**Published:** 2025-11-06

**Authors:** Sara Parola, Ilaria Colombo

**Affiliations:** Agostino Gemelli University Policlinic, Italy; ^1^Division of Breast Medical Oncology, Istituto Nazionale Tumori IRCCS, Fondazione G. Pascale, 80131 Napoli, Italy; ^2^Medical Oncology, Oncology Institute of Southern Switzerland (IOSI), Ente Ospedaliero Cantonale (EOC), 6500 Bellinzona, Switzerland

**Keywords:** bsAbs, solid tumors, immunotherapy, gynecological cancer, ovarian cancer, endometrial cancer, cervical cancer

## Abstract

Gynecological cancer remains one of the leading causes of mortality worldwide. Recent advances in genomic and molecular sequencing have significantly enhanced our understanding of the biological pathways that drive tumor progression and resistance to therapy. Targeted therapies, including monoclonal antibodies (mAbs), have revolutionized cancer treatment by selectively interfering with oncogenic proteins expressed on cancer cells. However, the long-term clinical benefit is often limited due to the emergence of drug resistance, frequently mediated by compensatory signaling pathways or immune escape mechanisms. To overcome these limitations, bispecific antibodies (bsAbs) represent an innovative class of therapeutic agents that have shown promising results across various medical fields. They have been developed to engage two distinct targets simultaneously, such as tumor antigens, immune effectors, or immunomodulatory checkpoints, thereby enhancing anti-tumor activity and reducing the risk of resistance. There are 17 bsAbs approved for clinical use in various countries, with numerous others currently in active development and over 600 bsAbs undergoing clinical trials worldwide. Among these, 11 have received FDA approval for the treatment of hematologic malignancies as well as solid tumors, including uveal melanoma, metastatic non-small cell lung cancer, small cell lung cancer, and biliary tract cancers. Although some studies have explored bsAbs in gynecological cancers, this area remains underdeveloped compared to other oncology fields. Most ongoing studies in this area are still in their early phases (phase I or phase II), and there is a need for optimization in terms of antibody design, efficacy, and safety profiles. Therefore, the purpose of this review is to present a comprehensive summary of the current research on bsAbs in gynecological cancers, with a focus on endometrial, cervical, and ovarian cancers. We will highlight ongoing clinical trials, discuss the mechanisms of action of these agents, and explore their potential benefits in enhancing treatment outcomes.

## Introduction

**Figure 1:**
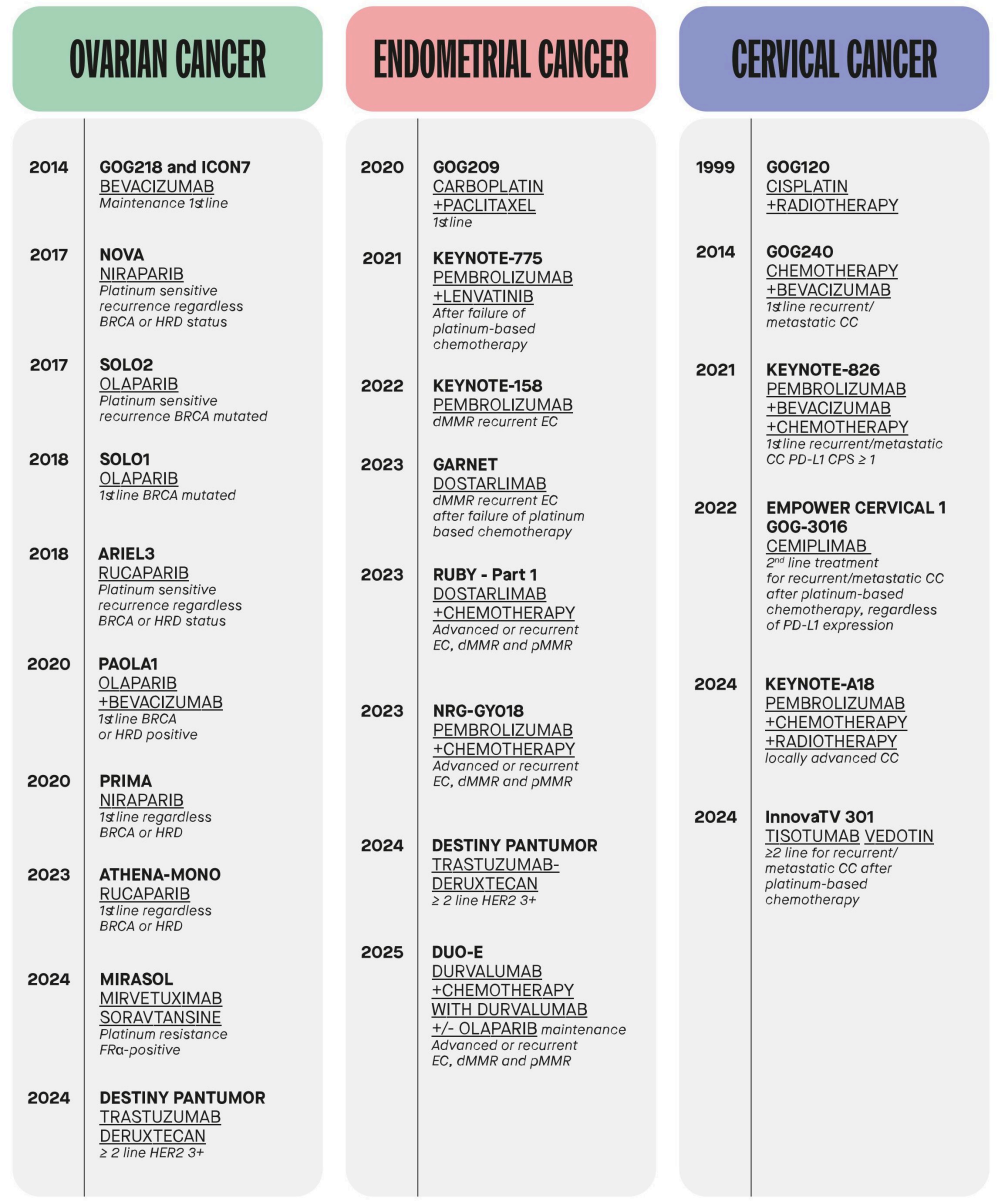
**Timeline of relevant clinical trials leading to therapeutic advances in ovarian, endometrial, and cervical cancer.** BRCA: breast cancer susceptibility gene; HRD: homologous recombination deficiency; FRα: folate receptor alpha; HER2: human epidermal growth factor receptor 2; EC: endometrial cancer; dMMR: deficient mismatch repair; pMMR: proficient mismatch repair; CC: cervical cancer; PD-L1: programmed death-ligand 1; CPS: combined positive score.

Gynecological malignancies represent a significant health burden worldwide. Among these, ovarian and endometrial cancers are the most prevalent and lethal in high-income countries [1]. Standard treatment paradigms have long depended on surgery, radiotherapy, and platinum-based chemotherapy [2]. However, relapses are frequent, even following initial clinical responses, due to tumor heterogeneity, late-stage diagnosis, and the development of intrinsic or acquired resistance to therapy [3]. The growing understanding of molecular mechanisms in these cancers has led to a shift toward precision oncology (Figure 1) [4].

A landmark example is the use of PARP inhibitors (PARPis), such as olaparib, niraparib, and rucaparib, in patients with breast cancer susceptibility gene (*BRCA*) mutations or homologous recombination deficiency (HRD), in ovarian cancer [5]. PARPis are now available to patients in both the first-line and recurrent platinum-sensitive disease settings, playing a crucial role in delaying disease recurrence [6–10]. The therapeutic landscape of gynecologic malignancies has evolved considerably with the introduction of targeted therapies, particularly anti-angiogenic agents like bevacizumab. In ovarian cancer, bevacizumab has demonstrated clinical benefit in combination with chemotherapy, improving progression-free survival (PFS) in various disease settings [11–14]. More recently, its combination with the PARPi olaparib has shown enhanced efficacy, especially in patients with HRD, offering a synergistic approach that further delays disease progression and improves survival [15]. In contrast, despite the success of immune checkpoint inhibitors (ICIs) in other solid tumors, their activity in epithelial ovarian cancer has been limited [16]. Antibody-drug conjugates (ADCs) have emerged as a powerful therapeutic platform, enabling the targeted delivery of cytotoxic agents to tumor cells expressing specific antigens [17]. Mirvetuximab soravtansine (MIRV), an ADC targeting folate receptor alpha (FRα), is now recognized as a standard of care treatment in platinum-resistant ovarian cancer, following the results of the phase III MIRASOL trial, which demonstrated improved response rates and survival outcomes compared to single agent chemotherapy [18, 19]. Other ADCs, such as trastuzumab deruxtecan (T-DXd), which targets human epidermal growth factor receptor 2 (HER2), are currently under investigation in gynecologic malignancies, showing promising preliminary efficacy [20].

The Cancer Genome Atlas (TCGA) in 2013 identified four distinct molecular subtypes of endometrial cancer: POLE ultra-mutated, microsatellite instability-high (MSI-H), copy number-low [also referred to as no specific molecular profile (NSMP)], and copy number-high (p53-abnormal). This classification, now commonly integrated into clinical practice, provides a more refined stratification of endometrial cancer beyond traditional histopathological parameters [21]. Currently, the identification of these molecular subtypes relies on a combination of immunohistochemistry and molecular assays, based on surrogate biomarkers. This stratification is increasingly used to guide prognosis and tailor therapeutic strategies. Among these subtypes, tumors with deficient mismatch repair (dMMR) or MSI-H are highly sensitive to ICIs [22]. These tumors exhibit high mutational burden and neoantigen load, which make them immunogenic and susceptible to programmed cell death protein 1 (PD-1)/programmed death-ligand 1 (PD-L1) blockade. In this context, anti-PD-1 antibodies, such as pembrolizumab and dostarlimab, have transformed treatment paradigms for dMMR or MSI-H tumors both in first line and in the recurrent setting in patients previously treated with platinum-based chemotherapy. The combination of pembrolizumab with lenvatinib has also extended clinical benefit to proficient mismatch repair (pMMR) tumors, as demonstrated in the KEYNOTE-775 study [23]. Dostarlimab, initially approved based on early-phase data, was later validated in the phase III RUBY trial [24]. Moreover, the DUO-E trial revealed that combining durvalumab (an anti-PD-L1 agent) with chemotherapy and olaparib might improve PFS, highlighting the potential of ICI-PARPi combinations [25].

In cervical cancer, despite preventive advances like human papillomavirus (HPV) vaccination and effective screening, advanced and metastatic disease remains a significant therapeutic challenge. The addition of pembrolizumab led to outcome improvement in different settings, as demonstrated by KEYNOTE-158, KEYNOTE-826, and KEYNOTE-A18 trials [26–28]. ADCs targeting tumor-specific antigens, such as tissue factor (tisotumab vedotin) and HER2 (T-DXd), have also demonstrated clinical benefit in heavily pretreated patients [20, 29].

**Figure 2:**
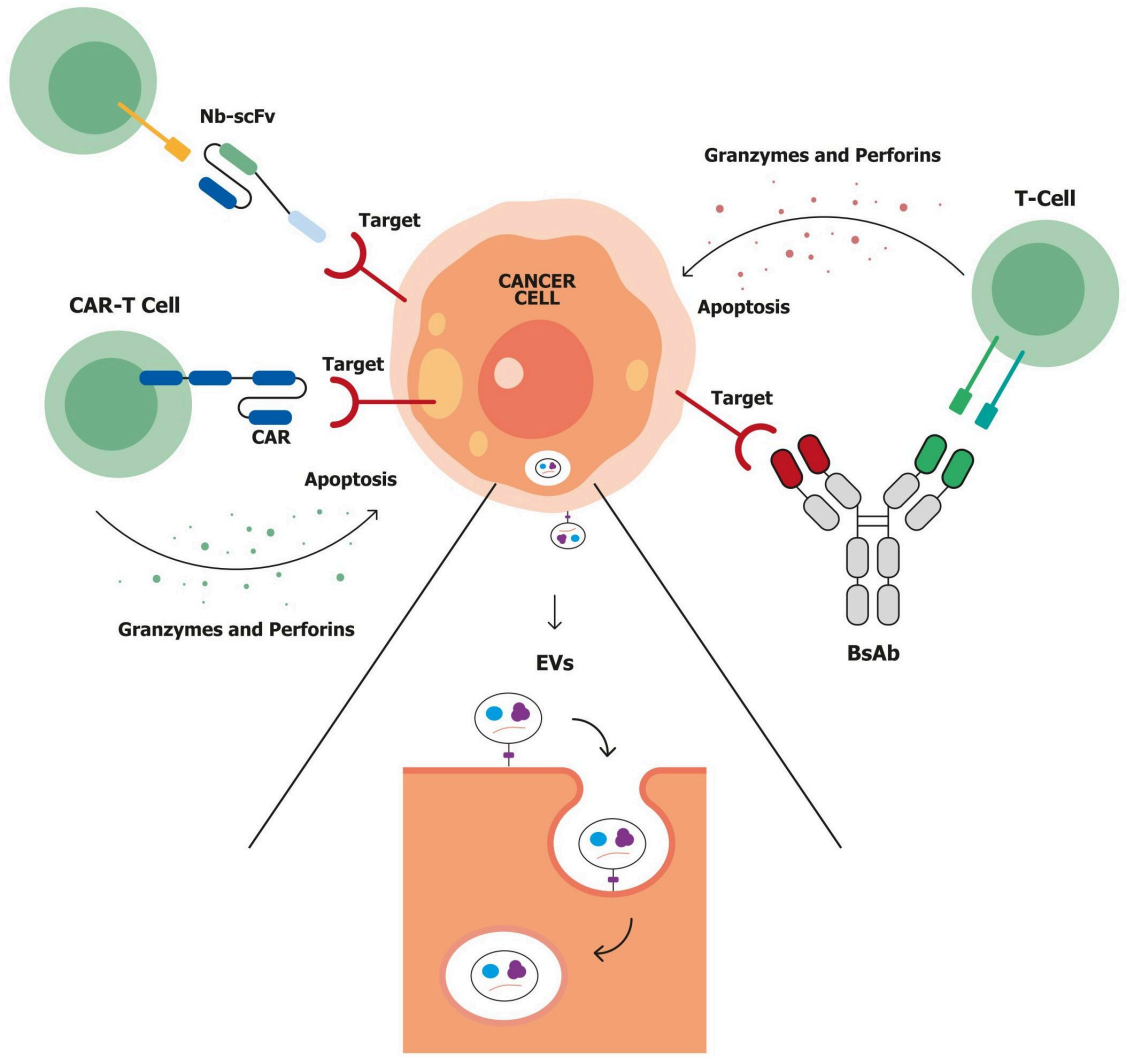
**Immune-mediated strategies for targeting tumor cells.** Possible immune-mediated strategies include chimeric antigen receptor T-cell (CAR-T), bispecific antibodies (bsAbs), nanobody-single-chain variable fragment (Nb-scFv), and extracellular vesicles (EVs) in tumor-immune interactions. **Left**: CAR-T cells are genetically engineered T-cells expressing CARs that recognize tumor-specific antigens. Upon antigen engagement, CAR-T cells release cytotoxic molecules (e.g., granzymes, perforins) and proinflammatory cytokines (e.g., IL-2, IFN-γ, TNF-α), leading to apoptosis of the cancer cell. **Top left**: Nb-scFv CAR constructs represent a next-generation CAR-T platform that incorporates nanobodies (single-domain antibodies) for improved tumor targeting, compact structure, and better tissue penetration. These also result in targeted tumor cell apoptosis. **Right**: bsAbs simultaneously engage tumor-associated antigens (TAAs) on cancer cells and CD3 receptors on T-cells, thereby redirecting T-cells toward tumor cells. This interaction promotes immune synapse formation and the release of granzymes and perforins, inducing apoptosis. **Bottom center**: EVs are membrane-bound nanoparticles released by tumor and immune cells within the tumor microenvironment (TME). They carry bioactive molecules, such as proteins, lipids, RNA, and immunomodulatory factors, which influence immune responses, promote therapy resistance, and support tumor progression.

Although substantial progress has been made in the treatment of gynecologic cancers, various intrinsic and acquired resistance mechanisms still contribute to therapeutic failure and disease progression [4]. Gynecologic malignancies exhibit diverse molecular mechanisms of resistance to chemotherapy, including altered drug transport, enhanced DNA repair, epigenetic dysregulation, and activation of survival pathways such as PI3K/AKT, MAPK, and mTOR [30]. In ovarian cancer, resistance is driven by overexpression of DNA repair proteins (e.g., BRCA1/2, RAD51), epigenetic alterations, and a hostile tumor microenvironment (TME) characterized by cancer-associated fibroblasts (CAFs), tumor-associated macrophages (TAMs), and extracellular matrix (ECM) remodeling [31]. In uterine cancer, resistance is often associated with *TP53* mutations, drug efflux pumps, and immune escape mechanisms such as PD-L1 upregulation [32, 33]. Cervical cancer adds complexity through HPV-mediated immune evasion, activation of DNA repair pathways, epithelial-to-mesenchymal transition (EMT), and enrichment of cancer stem cells [34]. These resistance pathways not only influence prognosis but also represent critical targets for novel therapies, including targeted agents and immunotherapies aimed at overcoming drug resistance and improving patient outcomes [4]. The emergence of novel biotechnological tools and genetic engineering has enabled the development of a new generation of innovative therapeutics (Figure 2).

These include various antibody-based platforms such as the fragment antigen-binding (Fab), single-chain variable fragment (scFv), and single-domain antibodies (sdAbs), also known as nanobodies (Nbs) [35]. Following the success of monoclonal antibodies (mAbs), research has increasingly focused on next-generation molecules with enhanced capabilities. Among these, Nbs have attracted significant attention due to their small size, high stability, and ability to penetrate solid tumors effectively [36]. Another innovative platform involves extracellular vesicles (EVs), which naturally carry proteins, lipids, and nucleic acids and can be engineered for targeted drug delivery. They offer excellent biocompatibility, the ability to cross biological barriers (e.g., the blood-brain barrier), and extended circulation times due to their expression of “don’t eat me” signals. Challenges remain, including EV heterogeneity, the need for standardized production protocols, and optimization of cargo-loading strategies for effective drug delivery [37]. Building on the evolution of mAbs, bispecific antibodies (bsAbs) represent another significant advancement [38]. These molecules can simultaneously bind two distinct targets, combining the specificities of two antibodies into a single construct. This dual-targeting capability enables bsAbs to mediate therapeutic effects more effectively than conventional mAbs, offering a versatile and powerful mechanism of action [39]. While bsAbs and Nbs are inherently active through antigen recognition, EVs serve primarily as delivery vehicles and must be loaded with functional cargo. Despite their therapeutic potential, however, bsAbs face several limitations, including immunogenicity, manufacturing complexity, and suboptimal tumor penetration. In contrast, the smaller size of Nbs and EVs allows for more efficient infiltration into solid tumors, particularly within the challenging environment of the TME [35]. Notably, some bsAbs are even engineered by fusing two Nbs with a flexible peptide linker, effectively combining the benefits of both formats, enhancing specificity, reducing size, and improving tissue penetration [39]. Chimeric antigen receptor T-cell (CAR-T) treatment is an additional possible immunotherapy approach that involves engineering a patient’s T-cells to recognize and attack cancer cells. These treatments also use scFvs for tumor recognition, using engineered T-cells to eliminate cancer cells directly [40]. While both bsAbs and CAR-T cells aim to activate the immune system against tumors, they do so in different ways. BsAbs act as connectors, physically linking a patient’s T-cells to cancer cells to trigger an immune response. CAR-T cells, by contrast, are autologous T lymphocytes that are genetically modified ex vivo to recognize and eliminate tumor cells independently, without the need for a bridging molecule [41]. Among the various emerging immunotherapeutic approaches investigated in gynecologic malignancies, bsAbs have recently gained considerable attention. This growing interest stems from the limited clinical success observed so far with other novel immunotherapies in these tumors [40]. Their ability to simultaneously engage two distinct targets, such as linking T-cells to tumor-associated antigens (TAAs) or blocking multiple oncogenic pathways, has shown encouraging results in early clinical studies [42, 43]. More than 100 bsAbs are currently under clinical investigation, primarily in oncology. Several have already received FDA approval, including amivantamab (non-small cell lung cancer), tebentafusp (uveal melanoma), blinatumomab (B-cell acute lymphoblastic leukemia), teclistamab (multiple myeloma), and glofitamab/mosunetuzumab (lymphoma) [44–49]. Encouraged by these successes, bsAb-based strategies are now being explored in gynecologic cancers. Ovarian cancers often overexpress a range of TAAs such as FRα, mucin 16 [MUC16; cancer antigen 125 (CA-125)], and mesothelin, making them ideal targets for bsAb development [50, 51]. Moreover, they may help bypass resistance mechanisms such as vascular endothelial growth factor (VEGF) mediated immunosuppression or T-cell exclusion by directly engaging immune cells at the tumor site [52]. In endometrial cancer, high tumor mutational burden (TMB) due to MSI-H or POLE mutations presents an opportunity to enhance immune surveillance through bsAbs targeting immune checkpoints or acting as T-cell engagers [53]. HER2 is also overexpressed in some endometrial cancers, especially serous subtypes. This makes HER2 a promising target for bsAb-based therapies [54]. In cervical cancer, immune evasion is often driven by persistent high-risk HPV infection. Upregulation of transforming growth factor-beta (TGF-β) signaling, frequent in HPV-driven tumors, contributes to an immunosuppressive TME, which bsAbs may help overcome [55]. These insights into the molecular and immunological basis of resistance and immune evasion highlight the potential of bsAbs to address key challenges in gynecologic cancers. By targeting multiple pathways simultaneously, they offer a unique opportunity to modulate the TME, enhance anti-tumor immunity, and improve therapeutic outcomes across diverse patient populations.

This review aims to provide a comprehensive overview of the current clinical landscape of bsAbs in gynecologic cancers, examining their mechanisms of action, therapeutic targets, safety profiles, and the most relevant ongoing clinical trials.

## Structure of bsAbs

**Figure 3:**
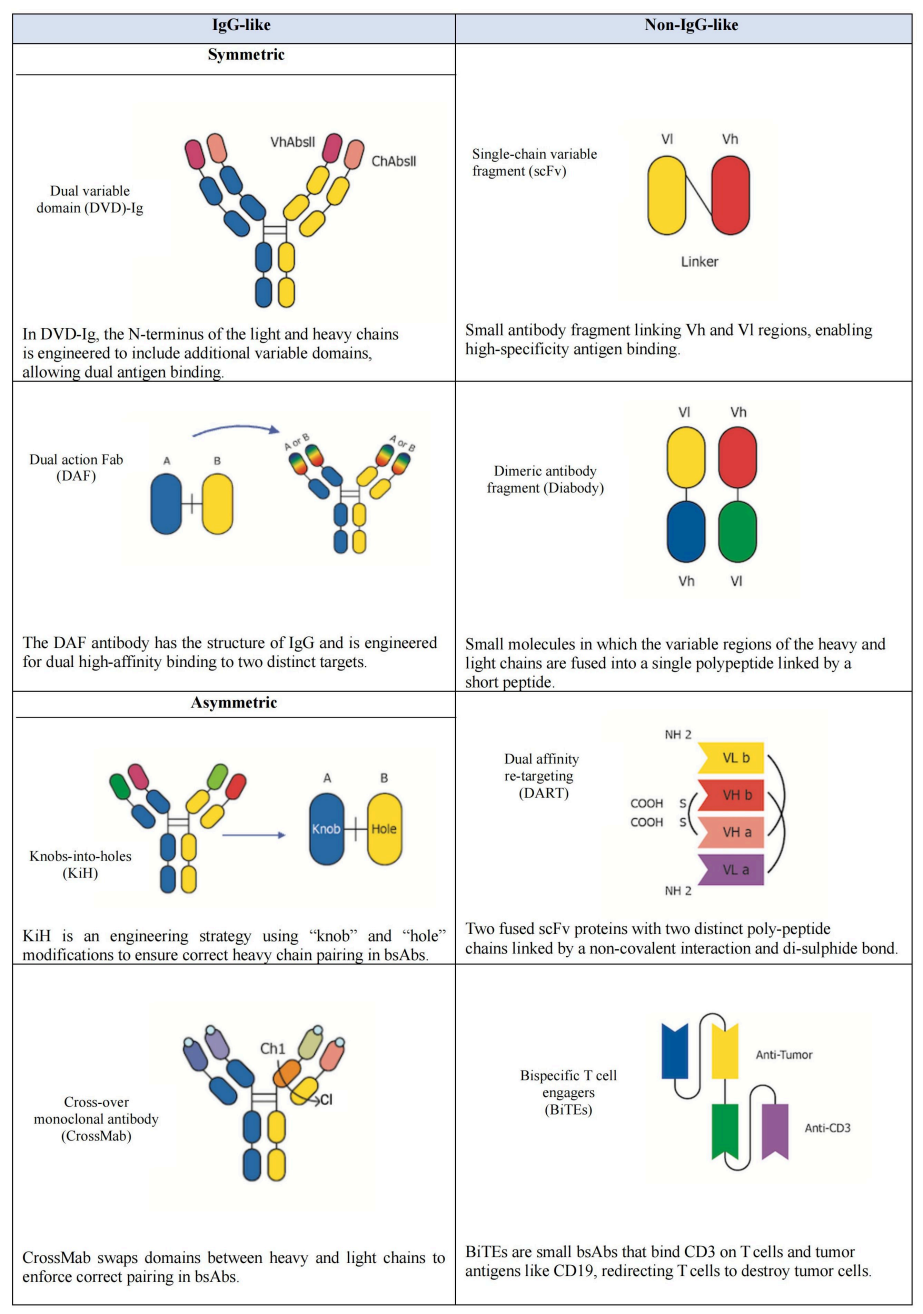
**Structure of selected types of bispecific antibodies (bsAbs).** This Figure illustrates a subset of the classification of engineered bsAbs, broadly categorized into “IgG-like” and “non-IgG-like” structures based on their molecular architecture. The “IgG-like” formats (left column) largely retain the bivalent, four-chain structure of a conventional IgG, including dual variable domain (DVD)-Ig, dual action fragment antigen-binding (DAF), knobs-into-holes (KiH), and cross-over monoclonal antibody (CrossMab). These designs are often developed to achieve bispecificity while maintaining a longer serum half-life. The “non IgG-like” formats (right column) represent smaller, more modular constructs that diverge significantly from the full IgG structure. These include single-chain variable fragment (scFv), dimeric antibody fragment (Diabody), dual affinity re-targeting (DART), and bispecific T-cell engagers (BiTEs). These smaller formats can offer advantages such as improved tissue penetration and simplified manufacturing processes. Each distinct structural design allows for the simultaneous binding of two different antigens or epitopes, enabling a wide range of novel therapeutic applications. VhAbsll: variable heavy antibody; ChAbsll: constant heavy antibody; Vl: variable regions of the light (chain); Vh: variable regions of the heavy (chain); Ch1: the first constant heavy domain; Cl: constant light domain; CD3: cluster of differentiation 3. NH2: amine group; COOH: carboxyl group; S-S: disulfide bond.

BsAbs are complex molecules that integrate two distinct heavy chains and two distinct light chains, allowing them to bind two different targets simultaneously [56]. Depending on their structural design, bsAbs can be symmetric or asymmetric, with asymmetry typically arising from differences in the Fv regions. Structurally, bsAbs can be classified into two main categories: non-IgG-like and IgG-like molecules, depending on the presence or absence of the fragment crystallizable (Fc) region, which significantly influences their functional properties (Figure 3) [57].

Fc-containing bsAbs (IgG-like molecules) are characterized by longer half-life and the ability to trigger immune cell effector functions, such as antibody-dependent cell-mediated cytotoxicity (ADCC), complement-dependent cytotoxicity (CDC), and antibody-dependent cellular phagocytosis (ADCP) [58]. The Fc region of IgG-based bsAbs promotes immune effector actions that contribute to anticancer activity by interacting with CD16 (FcγRIIIa) on natural killer (NK) cells [59]. However, these Fc-related processes may induce cytokine release syndrome (CRS) and nonspecific immune cell activation [60]. Moreover, researchers have introduced subcategories of symmetric (IgG-like) and asymmetric (IgG-modified) bsAbs to refine the classification of Fc-containing bsAbs based on their distinct structural characteristics [61]. Symmetrical bsAbs are derived from the fusion of identical polypeptide chains or paired light and heavy chains. While these closely resemble natural mAbs in many respects, they differ in molecular size, structure, stability, and solubility. In these molecules, the variable regions of the light (Vl) and heavy (Vh) chains are extended by the addition of an extra variable domain. This additional domain is connected to the original Vl or Vh domain via a specific linker sequence, creating a tandem variable structure. This structure yields a bsAb with four antigen recognition sites, enabling each Fab region to bind to two distinct targets [62]. Methods such as dual variable domain (DVD) and two-in-one can be used to construct IgG-like bsAbs. The DVD structure connects the Vl and Vh domains of an additional antibody at the N-terminus of the standard IgG antibody’s light chain and heavy chain, respectively. This design creates a DVD-Ig with two binding sites for each antigen. The DVD-Ig bsAb has the same Fc region as the standard mAbs and can be produced using established antibody manufacturing technologies [63]. Another effective strategy is a two-in-one antibody, where a single antibody is designed to bind to two different targets through a single binding site, and it is also known as dual action Fab (DAF). This method also preserves a symmetric antibody structure, ensuring stability and consistent pharmacokinetics while enabling dual antigen targeting [64]. Unlike traditional antibodies, which have a symmetrical structure, asymmetric antibodies break this symmetry to enable heterodimerization of their heavy chains. Developing asymmetric antibodies involves designing molecules with two distinct heavy chains, enabling them to bind to different antigens. This is achieved by modifying the Fc region to allow the pairing of two complementary heavy chains. Several methods are employed to achieve this asymmetric configuration [65]. The knobs-into-holes (KiH) approach is a widely used method, ensuring correct heavy chain pairing by introducing complementary structural modifications: a “knob” on one heavy chain and a corresponding “hole” on the other. This design promotes the formation of heterodimers, essential for maintaining specificity while preserving the overall IgG-like structure [66]. The cross-over mAb (CrossMab) technology enhances correct pairing by exchanging domains between the heavy and light chains, thereby reducing the likelihood of mispairing. Unlike the KiH approach, which modifies the Fc region to promote heterodimerization, CrossMab swaps the constant light domain (Cl) and the first constant heavy domain (Ch1) domains in one Fab domain of the bsAb while leaving the other Fab domain unchanged. Since the structures of Cl and Ch1 are highly similar, this interchange has minimal impact on the antibody’s function. Furthermore, the modified light chain is less likely to mismatch with the heavy chain of another normal Fab domain, ensuring proper pairing and maintaining the antibody’s stability and functionality [67]. Non-IgG-like bsAbs (Fc-free molecules) represent a group of constructs that lack the Fc region, including Fabs region, scFvs, and sdAbs. Fc-free bsAbs generally exhibit superior biodistribution within tumor tissues, higher potency, and a lower incidence of immune-related adverse events (irAEs). However, these bsAbs typically require continuous intravenous infusion or structural modifications to extend their half-life [68]. One common strategy to get around these issues is to use scFvs, in which the variable parts of the heavy and light chains are fused into a single polypeptide joined by a short peptide [69]. These scFvs can be combined to create bispecific structures that can bind to two distinct antigens simultaneously. One well-known example is the dimeric antibody fragment (Diabody), a non-covalent dimer created by connecting two scFvs with short linkers. It is a bsAb that detects two antigens and is compact and stable [70]. Dual affinity re-targeting (DART) molecules represent another innovative format within Fc-free bsAbs. DARTs consist of two variable domains from different antibodies, engineered to improve stability and target recognition by using a disulfide linkage to stabilize the heterodimer covalently. This design allows DART molecules to effectively and specifically engage two distinct targets [71]. Additionally, bispecific T-cell engagers (BiTEs) are a specialized class of non-IgG-like bispecific, composed of two scFvs, one targeting a TAA and the other targeting CD3 on T-cells. This structure facilitates the direct engagement of T-cells with tumor cells, thereby enhancing the immune response [72, 73]. Bispecific killer cell engagers (BiKEs) and trispecific killer engagers (TriKEs) extend the concept further by targeting not just CD3 but also NK cell receptors, such as CD16. This approach directs both T-cells and NK cells towards tumor cells, potentially increasing therapeutic efficacy by simultaneously engaging multiple types of immune effector cells [74, 75].

## Mechanisms of action of bsAbs

**Figure 4:**
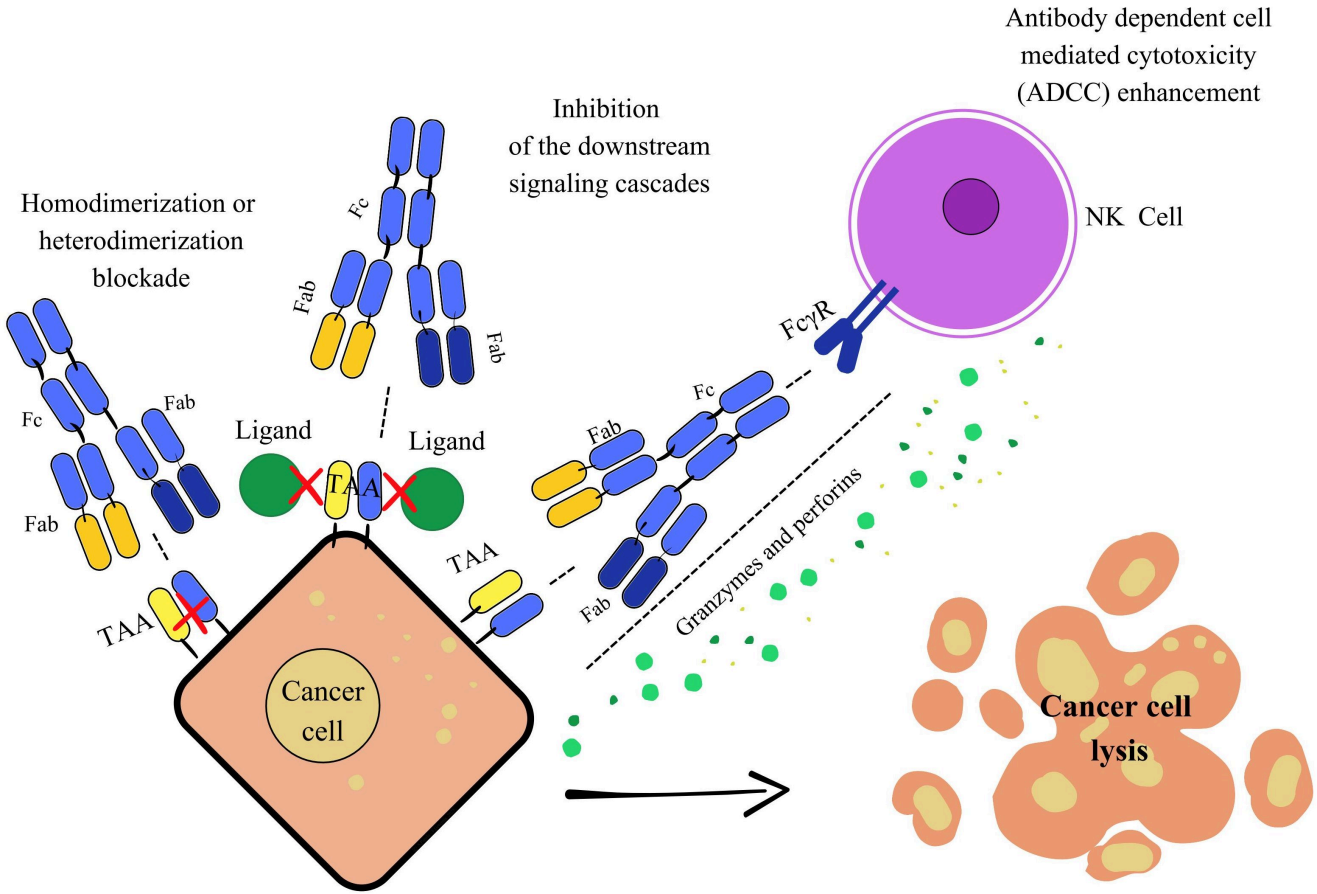
**Mechanism of action through signaling pathway blockade.** This image illustrates three key mechanisms of action for bispecific antibodies (bsAbs) against cancer cells. On the left, blocking dimerization and downstream signaling: The bsAbs simultaneously bind to two distinct targets on the tumor cell, preventing their interaction and dimerization. This blocks downstream signaling pathways essential for tumor growth and survival. In the middle, inhibition of downstream signaling cascades: By blocking dimerization or binding to two specific sites, the bsAbs can prevent growth and survival signals from being transmitted inside the tumor cell. On the right, enhancing antibody dependent cell mediated cytotoxicity (ADCC): The bsAbs bridge a tumor cell (via a TAA) and an immune effector cell (via an activating receptor like FcγR). This proximity activates the immune cell, leading to the release of cytotoxic molecules that cause the cancer cells’ lysis. Fc: fragment crystallizable; Fab: fragment antigen-binding; TAA: tumor-associated antigen; FcγR: Fc-gamma receptor; NK: natural killer.

BsAbs represent a versatile approach to cancer therapy. They simultaneously target multiple antigens or epitopes, enhancing immune activation [76]. One of the primary mechanisms of these drugs involves blocking two distinct antigens or different epitopes of the same antigen within key oncogenic pathways. Cancer cells express a wide range of TAAs, which bsAbs can target through multiple strategies. They can disrupt receptor-ligand interactions, inhibiting downstream signaling cascades essential for tumor growth, or induce receptor internalization, preventing receptor homodimerization or heterodimerization, thereby suppressing angiogenesis and proliferation. Additionally, IgG-based bsAbs with an Fc region can engage innate immune mechanisms such as ADCC and ADCP, further promoting tumor cell apoptosis. Dual-TAA targeting by bsAbs also enhances drug selectivity, ideally reducing off-target toxicities (Figure 4) [77].

**Figure 5:**
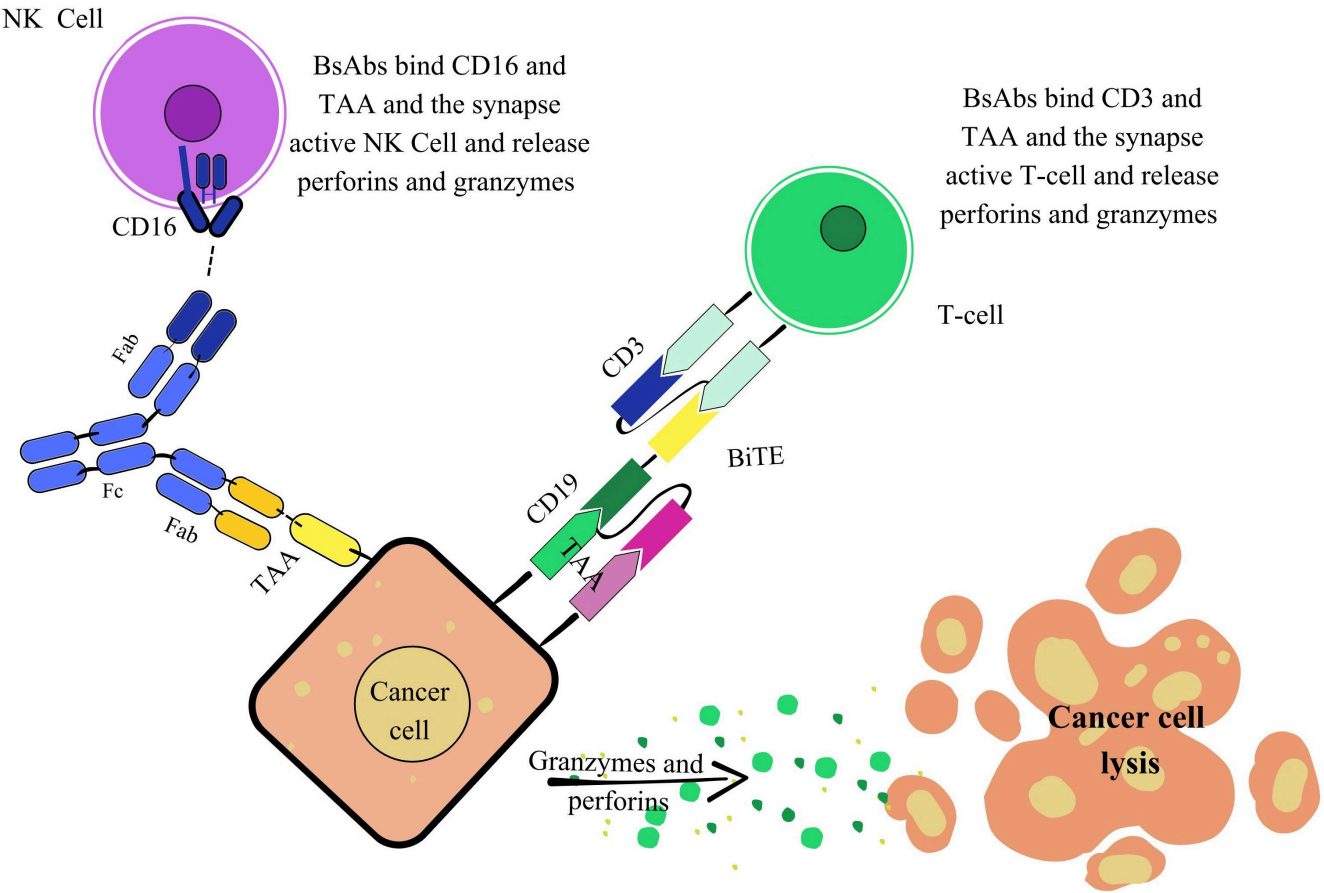
**Mechanism of action of immune cell engagers (ICEs).** The Figure illustrates strategies involving natural killer (NK) cells and T-cells. NK cell engagement: This mechanism describes how a bsAb recruits NK cells to induce tumor cell lysis. The bsAb features two fragments antigen-binding (Fabs): one targets CD16 (FcγRIIIa) on NK cells, and the other binds a tumor-associated antigen (TAA) on the cancer cell. This dual engagement forms an immunological synapse, activating the NK cell. The activated NK cell then releases perforins and granzymes, which are delivered to the cancer cell to initiate its lysis. T-cell engagement [bispecific T-cell engager (BiTE)]: This mechanism highlights the potent redirection of T-cell cytotoxicity against cancer cells mediated by BiTEs. A BiTE, typically a simplified bsAb format, often devoid of an Fc region, is engineered to bridge a T-cell and a cancer cell. They feature one binding domain for CD3 on the T-cell and another for a TAA (e.g., CD19) on the cancer cell. This forces an MHC-independent interaction, leading to potent T-cell activation. Activated T-cells then release perforins and granzymes, precisely delivered to induce efficient cancer cell lysis. bsAbs: bispecific antibodies; Fc: fragment crystallizable; CD3: cluster of differentiation 3.

Another crucial function of bsAbs is immune cell engager (ICE), where they bridge immune effector cells and tumor cells to trigger a potent cytotoxic response. Most ICEs are trans-binding bsAbs, typically composed of two linked scFvs derived from different mAbs. One binding site targets a TAA on cancer cells, while the other engages an immune receptor such as CD3 on T-cells, CD16 on NK cells, or CD64 on phagocytic cells. This dual binding facilitates the formation of an artificial immunological synapse, promoting T-cell proliferation, granzyme and perforin release, and selective tumor cell destruction. Unlike conventional immune responses, this mechanism is major histocompatibility complex (MHC)-independent, allowing bsAbs to be effective across a wide range of tumor types (Figure 5) [78, 79].

**Figure 6:**
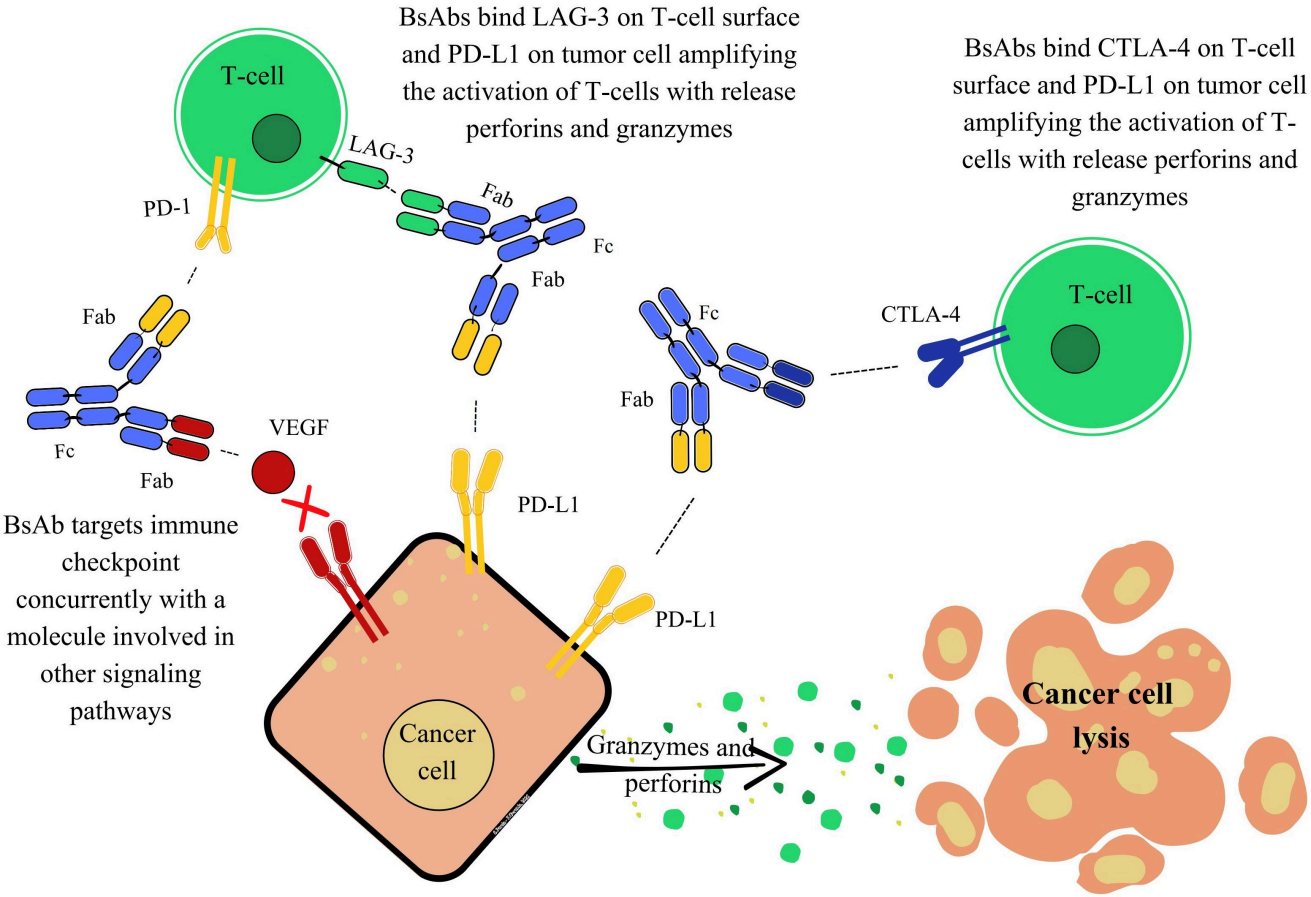
**Mechanisms of action of bsAbs targeting the immune checkpoint.** The Figure presents three distinct mechanisms. On the left, dual targeting of immune checkpoint and signaling pathway inhibition: This mechanism features a bsAb that targets an immune checkpoint molecule, programmed cell death protein 1 (PD-1), on the T-cell concurrently with a molecule involved in other signaling pathways, specifically vascular endothelial growth factor (VEGF) on the cancer cell. By simultaneously blocking PD-1, the bsAb aims to relieve T-cell inhibition, while also neutralizing VEGF to inhibit angiogenesis or other pro-tumor signaling. This dual action aims to amplify T-cell activation and directly hinder tumor growth pathways, contributing to cancer cell lysis. In the middle, immune checkpoint inhibition via LAG-3 and programmed death-ligand 1 (PD-L1) engagement: A bsAb is shown simultaneously binding to LAG-3 on the T-cell surface and PD-L1 on the cancer cell. This dual engagement blocks the inhibitory LAG-3 pathway and potentially the PD-1/PD-L1 axis by steric hindrance or recruitment. The outcome is the amplification of T-cell activation, leading to the release of perforins and granzymes, and subsequent cancer cell lysis. On the right, immune checkpoint inhibition via cytotoxic T-lymphocyte-associated protein 4 (CTLA-4) on the T-cell and PD-L1 on the tumor cell. By simultaneously binding both targets, this bsAb aims to counteract CTLA-4’s inhibitory effect on T-cell activation and potentially block PD-L1’s suppressive signals. This results in amplified T-cell activation, release of perforins and granzymes, and ultimately cancer cell lysis. bsAbs: bispecific antibodies; LAG-3: lymphocyte activation gene 3; Fab: fragment antigen-binding; Fc: fragment crystallizable.

Beyond direct tumor targeting and immune redirection, bsAbs also play a crucial role in ICI. By simultaneously targeting inhibitory receptors such as PD-1, PD-L1, cytotoxic T-lymphocyte-associated protein 4 (CTLA-4), lymphocyte activation gene 3 (LAG-3), or T-cell immunoreceptor with Ig and ITIM domains (TIGIT), bsAbs counteract the immunosuppressive signals that hinder T-cell activation, particularly within the TME. Unlike combinations of mAbs, they can selectively engage tumor-infiltrating lymphocytes (TILs) through avidity-mediated targeting, reducing systemic toxicity while enhancing immune responses. Moreover, bsAbs can redirect and activate immune cells by interacting with antigens involved in signaling pathways related to tumor development, angiogenesis, metastasis, and proliferation. This dual functionality is exemplified by their ability to combine VEGF blockade in the TME with immunomodulation via PD-1 inhibition [80]. Additionally, there is growing interest in dual immune checkpoint blockade strategies that target immunosuppressive pathways, such as the simultaneous modulation of TGF-β and PD-1/PD-L1 (Figure 6) [81, 82].

**Figure 7:**
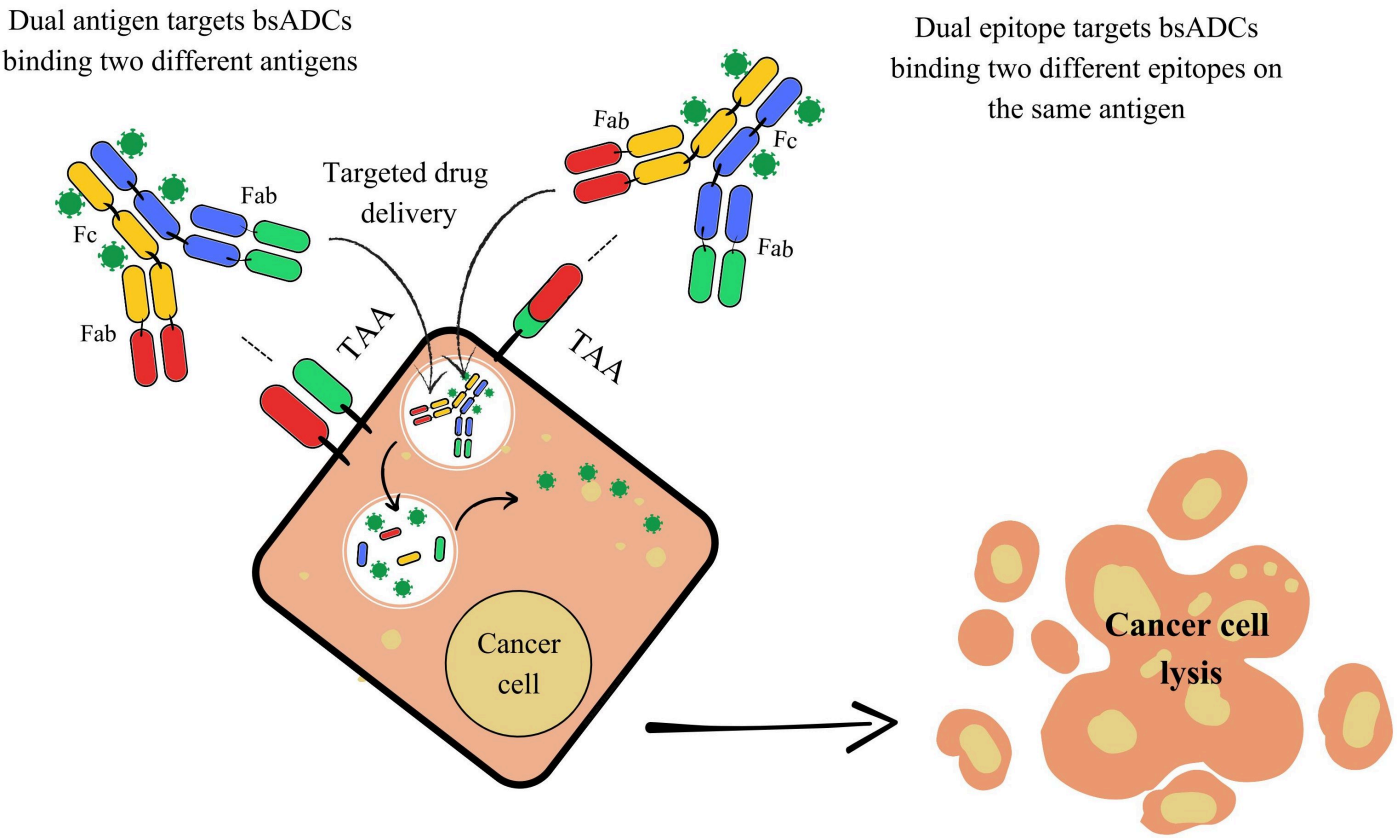
**Mechanism of action of bispecific antibody-drug conjugates (bsADCs).** This Figure illustrates two distinct bsADC targeting strategies for selective drug delivery and cancer cell killing. On the left, dual antigen target bsADCs are designed to bind two different tumor-associated antigens (TAAs) expressed on the same cancer cell. This dual-antigen binding increases tumor selectivity, particularly in heterogeneous tumors, by requiring co-expression of both TAAs for effective targeting. Upon binding, the bsADC is internalized into the cancer cell, leading to intracellular release of the conjugated cytotoxic payload and subsequent cell lysis. On the right, dual epitope target bsADCs bind two distinct epitopes on a single TAA. This strategy enhances binding avidity and specificity, improving internalization efficiency and drug delivery. Once inside the cell, the cytotoxic agent is released, resulting in targeted cancer cell death. Fc: fragment crystallizable; Fab: fragment antigen-binding.

Moreover, the conjugation of bsAbs to a linker-payload complex has led to the development of bsAb drug conjugates (bsADCs). These molecules combine the dual-targeting capability of bsAbs with the cytotoxic potential of ADCs, improving tumor cell destruction while reducing off-target toxicity. Compared to traditional ADCs, bsADCs enhance specificity and drug internalization, leading to greater tumor eradication and a higher therapeutic index. They can be categorized based on their binding patterns: They can either bind two different antigens simultaneously (dual-antigen binding mode) or target two distinct epitopes on the same antigen (dual-epitope binding mode). Advancements in ADC technology, such as improved lysosomal shuttling and the integration of innovative payloads including toxins, radioisotopes, and cytokines, further broaden the therapeutic potential of bsAbs in cancer treatment (Figure 7) [83, 84].

## Clinical evidence of bsAbs in gynecological cancer

BsAbs are being increasingly studied for gynecological malignancies as novel therapeutic agents to expand treatment options and improve patient outcomes.

### Ovarian cancer

In ovarian cancer, many bsAbs under evaluation act as ICE or ICI, enhancing the anti-tumor immune response (Table 1).

**Table 1:** Summary of main clinical trials investigating bsAbs in ovarian cancer.

Drug name	Target	Setting	Phase	*n*	Efficacy	TEAEs/TRAEs/irAEs G ≥ 3* (in at least 5% of pts) and any grade CRS/IRR/ICANS** (%)
ICE mechanism						
Ubamatamab (REGN4018) [85]	MUC16 × CD3	PROC 1–3 lines	1	78	ORR: 14.3% (95% CI: 5.4–28.5) DCR: 57.1% (95% CI: 41–72.3)	TEAEs G ≥ 3 anemia 24.4% neutropenia 7.7% pain 23.1% Any grade CRS 74.4% ICANS 1.3% IRR NR
Ubamatamab (REGN4018) + cemiplimab [86]	MUC16 × CD3 + antibody anti-PD-1	PROC 1–3 lines	1	35	ORR: 18.2% (95% CI: 5–40)	TEAEs G ≥ 3 anemia 23% pain 20% neutropenia 9% fatigue 9% hypo-phosphataemia 6% irAEs G ≥ 3 no events ≥ 5% Any grade CRS 68.6% ICANS 3% IRR NR
REGN5668 + cemiplimab [87]	MUC16 × CD28 + antibody anti-PD-1	PROC ≥ 1 line	1	28	ORR: 4%	TRAEs/irAEs G ≥ 3 no events ≥ 5% Any grade CRS 10.7% IRR 7.1% ICANS NR
Catumaxomab [88]	EpCAM × CD3	PROC ≥ 3 lines	2	32	mPuFI: 29.5 days (95% CI: 15–53) mOS: 111 days (95% CI: 66–140)	TRAEs G ≥ 3 vomiting 13% nausea 13% fatigue 13% ALP increased 9% pain 6% dehydration 6% Any grade CRS NR IRR/ICANS NR
ABBV-428 [89]	Mesothelin × CD40	PROC 1–3 lines	1	59 (all pts) 14 (ovarian cancer)	ORR: 0	TRAEs G ≥ 3 pericardial effusion 5% colitis 5% Any grade IRR 12% CRS 0% ICANS NR
JNJ-78306358 [90]	HLA-G × CD3	PSOC PROC > 2 lines	1	39 (all pts) 10 (ovarian cancer)	ORR: 0	TEAEs G ≥ 3 pain 10.3% anemia 5.1% lymphopenia 5.1% neutropenia 5.1% pneumonia 5.1% pulmonary embolism 5.1% ALT increased 5.1% hypertension 5.1% Any grade CRS 48.7% IRR/ICANS NR
Brenetafust +/– Gem or NP or PLD [91]	Gp100 × CD3	PROC ≤ 4 lines	1	47 (all pts) Monotherapy: 37 Combination-therapy: 16	Monotherapy: DCR: 58% mPFS: 3.3 mths (95% CI: 2.1–4.0) Combination-therapy: DCR: 69% mPFS: NA	TRAEs G ≥ 3 monotherapy a. no events ≥ 5% any grade a. CRS 57% b. IRR/ICANS NR TRAEs G ≥ 3 combination-therapy a. fatigue 6% b. ALT/AST increased 19%/13% any grade a. CRS 75% b. IRR/ICANS NR
ICI mechanism						
Vudalimab (XmAb20717) [92]	PD-1 × CTLA-4	PROC	1	110 (all pts) 20 (ovarian cancer)	ORR: 13% (all pts) 5% (ovarian cancer)	irAEs G ≥ 3 rash 16.4% AST/ALT increased 9.1% hyper-glycemia 4.5% Any grade IRR/CRS/ICANS NR
Cadonilimab (AK104) + platinum + taxane [93]	PD-1 × CTLA-4	PSOC Neoadjuvant setting	2	27	R0 resection rate: 76.2% ORR: 91.6%	TRAEs G ≥ 3 NA 12.5% type not specified irAEs G ≥ 3 no events ≥ 5% Any grade IRR/CRS/ICANS NR
Tebotelimab (MGD013) +/– margetuximab [94]	PD-1 × LAG-3	> 1 prior line	1	277 (all pts) Monotherapy: 40 (ovarian cancer) Combination: 7 (ovarian cancer)	Monotherapy: ORR 11% (4/36; 95% CI: 3–26) Combination: ORR 19% [all pts HER2+ regardless PD-L1 (14/72; 95% CI: 11–30)]	TRAEs G ≥ 3 monotherapy a. no events ≥ 5% irAEs G ≥ 3 no events ≥ 5% all grade a. IRR 7.4% b. CRS/ICANS NR Combination: TRAEs G ≥ 3 AST increased 5% irAEs G ≥ 3 no events ≥ 5% any grade a. IRR 7.1% b. CRS/ICANS NR
Ivonescimab (AK112/SMT112) [95]	PD-1 × VEGF	PROC ≥ 3 lines	1	51 (all pts) 19 (ovarian cancer)	ORR: 26.3%	TRAEs G ≥ 3 hypertension 13.7% ALT increased 5.2% irAEs G ≥ 3 no events ≥ 5% any grade a. IRR 7.8% b. CRS/ICANS NR

The Table provides a comprehensive overview of the main clinical trials evaluating bsAbs in ovarian cancer. Only studies with available clinical data in ovarian cancer are reported, with a focus on target specificity, study phase, treatment combinations, and preliminary efficacy or safety outcomes when available. *: Some trials reported treatment-related adverse events (TRAEs), other treatment-emergent adverse events (TEAEs), and immune-related adverse events (irAEs). Only grades ≥ 3 observed in more than 5% of patients are included in this Table; **: adverse events of special interest (AESIs), including cytokine release syndrome (CRS), infusion-related reaction (IRR), and immune effector cell-associated neurotoxicity syndrome (ICANS), are listed in the Table regardless of grade and even if occurring in less than 5% of patients. bsAbs: bispecific antibodies; *n*: number; G: grade; pts: patients; ICE: immune cell engager; MUC16: mucin 16; PROC: platinum-resistant ovarian cancer; ORR: objective response rate; CI: confidence interval; DCR: disease control rate; NR: not reported; PD-1: programmed cell death protein 1; EpCAM: epithelial cell adhesion molecule; mPuFI: median puncture-free interval; mOS: median overall survival; ALP: alkaline phosphatase; HLA-G: human leukocyte antigen-G; PSOC: platinum sensitive ovarian cancer; ALT: alanine aminotransferase; Gem: gemcitabine; NP: nab-paclitaxel; PLD: pegylated liposomal doxorubicin; mPFS: median progression-free survival; mths: months; NA: not available; AST: aspartate aminotransferase; ICI: immune checkpoint inhibitor; CTLA-4: cytotoxic T-lymphocyte-associated protein 4; LAG-3: lymphocyte activation gene 3; HER2: human epidermal growth factor receptor 2; PD-L1: programmed death-ligand 1; VEGF: vascular endothelial growth factor.

#### ICE mechanism

Ubamatamab (REGN4018) is a bsAb that targets MUC16, also known as CA-125, and CD3, thereby redirecting T-cells toward MUC16-expressing ovarian cancer cells. MUC16 is a high-molecular-weight, *O*-glycosylated glycoprotein that contributes to the formation of a protective mucous barrier on epithelial surfaces and plays a significant role in shaping the TME, influencing immune cell interactions, and potentially contributing to immune evasion. Preclinical data suggest that MUC16 can modulate immune responses by engaging immune cells, such as neutrophils and NK cells, potentially fostering an immunosuppressive environment [96]. In a phase I clinical study, 78 patients with recurrent ovarian cancer received ubamatamab at doses ranging from 0.3 to 800 mg without reaching a maximum tolerated dose. The median number of prior therapies was 4.5 (range 1–17). Among 42 patients who received at least one full dose ≥ 20 mg, the objective response rate (ORR) was 14.3% (95% CI: 5.4–28.5), with a disease control rate (DCR) of 57.1% (95% CI: 41–72.3) and a median duration of response (DoR) of 12.2 months. The most common treatment-emergent adverse events (TEAEs) were CRS, occurring in 74.4% of patients (all grade 1–2), and pain of all grading levels, reported in 87.2% of patients, particularly during the first two weeks of step-up dosing. Additionally, one case of immune effector cell-associated neurotoxicity syndrome (ICANS) was reported. Grade ≥ 3 TEAEs observed in > 5% of patients included anemia and neutropenia [85]. In a subsequent trial combining ubamatamab with the anti-PD-1 antibody cemiplimab, 35 patients with ovarian cancer (median 5 prior therapies) were treated. Ubamatamab was administered weekly (1–450 mg) with cemiplimab added after 4 weeks. Treatment durations were 11 and 12 weeks, respectively. Among 22 patients receiving both agents, ORR was 18.2% (95% CI: 5–40), median DoR 8.3 months (95% CI: 4.2–not estimable). 6- and 12-month PFS was 48% (95% CI: 26–67) and 24% (95% CI: 9–43). TEAEs were consistent with prior data. Grade ≥ 3 TEAEs included anemia, pain, and neutropenia, with one case of dose-limiting toxicity (DLT) due to neutropenia at 60 mg. CRS was the most frequently reported TEAE. Additionally, an episode of ICANS and an immune-mediated adverse reaction (imAR) were also reported. Notably, a case of grade 4 hemophagocytic lymphohistiocytosis occurred at the 450 mg dose level, raising safety concerns at higher doses. Most of the TEAEs occurred within the first 4 weeks of treatment, which corresponds to the monotherapy period with ubamatamab. In contrast, irAEs were observed following the addition of cemiplimab, suggesting a potential association with the combination phase [86]. Although ubamatamab has shown preliminary signs of anti-tumor activity in a heavily pretreated population, several important limitations remain. Notably, no correlation between MUC16 expression and clinical response has been reported to date. Additionally, safety concerns have emerged: a substantial proportion of patients experienced TEAEs, including CRS, pain, anemia, and neutropenia. Of particular concern was the occurrence of grade 4 hemophagocytic lymphohistiocytosis at higher dose levels. The absence of biomarker-guided stratification, especially regarding MUC16 expression, currently limits the ability to tailor treatment based on predictive markers. To address these gaps, a randomized phase II trial has been initiated, incorporating initial step-up dosing followed by dosing every three weeks. The trial includes exploratory endpoints such as the evaluation of baseline tumor MUC16 expression by immunohistochemistry and other biomarkers as potential predictors of response. Further understanding of ubamatamab’s efficacy, safety profile, and optimal patient selection for ovarian and endometrial cancer is expected from the ongoing trial [97]. Another MUC16-targeting agent currently under investigation is REGN5668 (R5668), a bsAb designed to simultaneously bind MUC16 on tumor cells and CD28 on T-cells, thereby providing a co-stimulatory signal to enhance T-cell activation in the TME. In a phase I dose-escalation study, intravenous R5668 was evaluated in combination with the anti-PD-1 mAb cemiplimab in patients with recurrent ovarian cancer. Patients received weekly R5668 at doses ranging from 0.3 to 300 mg, with cemiplimab (350 mg IV every 3 weeks) initiated between days 21 and 28. The combination was generally well tolerated, with most treatment-related adverse events (TRAEs) being grade 1–2, including fatigue, nausea, and pain. Only one patient experienced a grade ≥ 3 TRAE (fatigue), and no DLTs were reported. irAEs were identified in 2 patients (7%), including 1 case of grade 2 hyperthyroidism and 1 case of grade 2 hypothyroidism. However, a limitation is the inability to determine whether these events were related to cemiplimab or to the concurrently administered bsAb. Although early signs of clinical activity were observed, one patient at the 300 mg dose level demonstrated a confirmed partial response (PR) with a 59% reduction in target lesion size and a CA-125 response. Additionally, 21% of patients achieved stable disease [87]. A phase I/II is ongoing (NCT04590326).

Catumaxomab is a trifunctional bsAb that binds to epithelial cell adhesion molecule (EpCAM) on tumor cells and CD3 on T-cells. At the same time, its Fc region activates Fcγ receptors on accessory immune cells, including NK cells, dendritic cells, and macrophages. The antibody’s constant Fc region interacts with immune cell receptors to mediate immune effector actions. In epithelial malignancies like ovarian cancer, where EpCAM overexpression is associated with a poor prognosis, catumaxomab has demonstrated special relevance. By bridging tumor cells and T lymphocytes and recruiting accessory immune cells like macrophages and NK cells, catumaxomab induces tumor cell death through apoptosis and phagocytosis [98]. In the first randomized, open-label phase II/III trial (IP-REM-AC-01), catumaxomab combined with paracentesis demonstrated significant clinical benefit in treating malignant ascites from epithelial cancers. Among 258 patients with histologically confirmed epithelial cancers and EpCAM-positive tumor cells in ascitic fluid, those receiving catumaxomab plus paracentesis had a markedly prolonged median puncture-free survival (PuFS) compared to paracentesis alone [46 vs. 11 days; hazard ratio (HR) 0.2254; *p* < 0.0001]. This benefit was consistent across ovarian and non-ovarian cancer subgroups, as well as in patients with and without distant metastases. The median time to next therapeutic paracentesis was also significantly extended (77 vs. 13 days; HR 0.169; *p* < 0.0001), with the gastric cancer subgroup showing the greatest improvement (118 vs. 15 days). While median overall survival (OS) did not significantly differ in the overall population, a significant OS benefit was observed in the gastric cancer subgroup [99]. In another phase I/II trial (STP-REM-01), intraperitoneal administration of catumaxomab via 6-h infusions of doses ranging from 5 to 200 μg over 9 to 13 days significantly reduced ascites flow rate in 23 patients with malignant ascites secondary to advanced ovarian cancer. Notably, 22 patients did not require paracentesis between the final infusion and day 37. Based on this study, a dosing regimen of four infusions at 10, 20, 50, and 150 μg was recommended for subsequent clinical trials [100]. Clinical studies further demonstrated that intraperitoneal catumaxomab prolonged the PuFS from a median of 12 to 27.5 days and extended the time to ascites progression. Approximately 23% of patients reached the primary efficacy endpoint, with a median OS of 111 days. Safety analysis revealed frequent TRAEs, including nausea, vomiting, fever, fatigue, and abdominal pain. Grade 3 or higher toxicities were observed in half of the patients, and serious adverse events (SAEs) occurred in about one-quarter. CRS and transient elevations of liver enzymes were commonly reported but generally reversible. Treatment discontinuation due to toxicity occurred in 16% of patients, highlighting a notable limitation in tolerability [88]. A separate study evaluating catumaxomab as consolidation therapy post-relapse reported a median PFS of approximately 17 months, supporting its potential utility in this setting [101]. Despite promising clinical activity, catumaxomab presents notable limitations, particularly a high incidence of TRAE, which may compromise tolerability. Moreover, its efficacy is contingent on EpCAM expression, highlighting the need for standardized, validated testing methods to ensure appropriate patient selection. Catumaxomab was approved by the European Medicines Agency (EMA) on February 11, 2025, for the intraperitoneal treatment of malignant ascites in patients with EpCAM-positive tumors [102, 103]. This approval likely reflects its ability to reduce the frequency of paracentesis, a burdensome procedure for patients with advanced epithelial cancers and symptomatic ascites. Unlike most oncology trials that use PFS or OS as primary endpoints, catumaxomab studies prioritized PuFS, the time to next paracentesis, as a patient-centered outcome. While not widely adopted in oncology, this endpoint directly addresses symptom burden and quality of life. Future trials should consider integrating both symptomatic and oncologic endpoints to better capture the full clinical benefit.

Mesothelin is another target highly expressed in ovarian cancer. ABBV-428 combines mesothelin targeting with CD40 activation, enhancing the immune response against mesothelin-expressing ovarian tumor cells. CD40 is a key immune receptor that stimulates adaptive immunity and promotes tumor stroma destruction. In a phase I trial, it demonstrated an acceptable safety profile; however, no treatment responses were observed [89]. Although mesothelin is a promising therapeutic target in ovarian cancer, this drug failed to demonstrate clinical benefit. This may be partly due to the absence of patient selection based on mesothelin expression, which could have limited effective tumor targeting. This aligns with exploratory findings showing only a modest correlation between baseline mesothelin levels and PFS [104]. Moreover, CD40 agonism is highly context-dependent, requiring a functionally immunocompetent and inflamed TME to propagate immune activation. Ovarian cancer is often characterized by an immunosuppressive TME with low infiltration of activated T-cells and dendritic cells, potentially reducing the immunostimulatory capacity of CD40-based therapies. In this context, ABBV-428 may not have achieved sufficient intra-tumoral engagement or downstream immune activation to mediate tumor regression. This is supported by the absence of pharmacodynamic changes in key intratumoral markers, such as CD8+ T-cells, PD-L1+ cells, or immune-related gene expression following treatment. Interestingly, irAEs were observed and they did not correlate with therapeutic benefit, raising concerns about the therapeutic index of CD40 agonists in low-inflamed tumors. The discordance between toxicity and efficacy suggests that systemic immune activation may occur even in the absence of effective tumor-localized immunostimulation [105]. Ongoing trials are now exploring alternative strategies, including NI-1801, a bsAb targeting mesothelin and CD47, currently under investigation as monotherapy and in combination with paclitaxel or pembrolizumab (NCT05403554).

A promising area of research involves XmAb541 and CTIM-76, bsAbs targeting claudin-6 (CLDN6), a tight junction protein that is selectively expressed on cancer cells with little to no presence in normal healthy tissue [106]. In gastric cancer, CLDNs, particularly CLDN6, are being actively targeted in an ongoing study (NCT04503278). Additionally, there is increasing interest in CLDN18.2 as a potential therapeutic target. Recent studies have demonstrated the efficacy of CLDN18.2-targeting agents, such as zolbetuximab, in combination with chemotherapy for CLDN18.2-positive metastatic gastric cancer, underscoring the growing potential of CLDNs as valuable targets in the treatment of this difficult-to-treat cancer. The promising results in gastric cancer further sustain opportunities for CLDN-targeting therapies in other tumor types [107]. Two-phase Ia/Ib open-label, dose-escalation, and expansion studies are currently recruiting participants to evaluate the safety and efficacy of XmAb541 and CTIM-76, a humanized T-cell-engaging bsAbs targeting CLDN6, in subjects with platinum-resistant ovarian cancer and other advanced CLDN6-positive solid tumors, including testicular and endometrial cancers (NCT06276491, NCT06515613). JNJ-78306358 is another drug that redirects T-cells through CD3 to target and kill tumor cells expressing human leukocyte antigen-G (HLA-G). HLA-G expression has been observed in various malignancies and is strongly associated with tumor immune escape, metastasis, and poor prognosis [108]. Despite a strong biological rationale for targeting HLA-G, the only phase I trial of JNJ-78306358 showed limited clinical activity, with no ORR observed. The agent demonstrated pharmacodynamic effects, including cytokine induction and T-cell activation, but was also linked to CRS-related toxicities. These AEs ultimately restricted dose escalation and prevented progression to phase II dose expansion [90].

Brenetafusp, an immune-mobilizing monoclonal T-cell receptor against cancer (ImmTAC) bsAbs targeting an HLA-A*02:01-presented preferentially expressed antigen in melanoma (PRAME) antigen and CD3, has shown early signs of activity in solid tumors. PRAME is a cancer-associated antigen highly expressed in various tumors but with low or absent expression in normal tissue, making it an attractive target for immunotherapy. ImmTACs utilize engineered T-cell receptors (TCRs) with ultra-high affinity for tumor-specific antigens, allowing for the targeting of intracellular proteins presented on the surface of cancer cells via HLA molecules. The CD3-binding domain recruits and activates T-cells, driving a potent immune response specifically against PRAME-expressing tumor cells. Brenetafusp has been evaluated both as a monotherapy and in combination with chemotherapy agents (gemcitabine, nab-paclitaxel, and pegylated liposomal doxorubicin). The DCR was 58% in the monotherapy group and 69% in the combination chemotherapy group. Molecular response, assessed by circulating tumor DNA (ctDNA) reduction, was observed in 24% of monotherapy patients and 90% of combination chemotherapy patients, with responses trending toward longer PFS and OS. Among 28 monotherapy patients evaluable for tumor cell fraction (TCF), those with TCF ≥ median had a higher DCR (80%) compared to patients with TCF < median (38%) and a longer median PFS [3.7 vs. 2.2 months; HR 0.39 (95% CI: 0.15–0.97)]. Notably, this study is one of the few to evaluate molecular response via ctDNA reduction. Compared to other bsAbs previously studied, brenetafusp appears to have a more favorable toxicity profile, with no reported cases of grade 3 CRS. AEs observed in the combination therapy group were predominantly related to chemotherapy-associated toxicities. Given that this is a phase I trial, further studies are required to confirm these findings and better characterize the clinical benefit and safety profile of brenetafusp [91].

PF-07260437, targeting B7-H4 and CD3, is under investigation for its potential in patients with platinum-resistant ovarian cancer. B7-H4, an immune checkpoint molecule overexpressed in several cancers, including ovarian cancer, suppresses T-cell activity and contributes to immune evasion. This agent aims to enhance anti-tumor immunity by simultaneously engaging CD3 to activate T-cells. Similarly, XmAb808, another bsAbs, targets B7-H3, a TAA overexpressed in various cancers, and CD28, a co-stimulatory receptor on T-cells. By selectively activating T-cells in the presence of B7-H3-expressing tumor cells, XmAb808 seeks to enhance the immune response within the TME while minimizing systemic toxicity. A phase I first-in-human dose-escalation and expansion study has evaluated the safety, tolerability, pharmacokinetics, and activity of XmAb808 in combination with pembrolizumab in selected advanced solid tumors (NCT05585034). These agents aim to overcome T-cell exhaustion and promote costimulatory signaling in TME, but the balance between immune activation and systemic toxicity is to be carefully monitored.

#### ICI mechanism

The preliminary results from the phase I study of XmAb20717, a dual PD-1/CTLA-4 checkpoint inhibitor, showed an ORR of 13%, which may appear modest. However, the enrolled population was heavily pretreated and heterogeneous, including patients with various advanced solid tumors and a median of multiple prior therapies, factors that typically limit ORR. Pharmacodynamic assessments confirmed biological activity through robust CD8+ and CD4+ T-cell proliferation and increased intra-tumoral immune infiltration. Despite these encouraging signs, irAEs were significant, highlighting the need for careful dose optimization to balance efficacy and safety. As a phase I trial primarily focused on safety and dose determination, these findings remain preliminary [92]. A phase II study evaluating XmAb20717 in gynecologic and genitourinary cancers is currently active but not recruiting. It will be essential to better define the therapeutic potential and safety profile of this agent (NCT05032040). In the neoadjuvant setting, cadonilimab (AK104), a tetravalent bsAbs targeting PD-1 and CTLA-4, is being evaluated in combination with neoadjuvant chemotherapy for advanced-stage ovarian cancer. This is the first bsAb to be explored in this setting of disease. In a recent study enrolling 27 patients, most had high-grade serous carcinoma (95.8%) and FIGO stage IV disease (83.4%). Interval debulking surgery was performed in 79.2% of patients, achieving an R0 resection rate of 76.2%. ORR was 91.6%, with 8.3% complete responses. PFS and OS data are not yet mature. TRAEs occurred in 62.5% of patients, with grade ≥ 3 events in 12.5%. The most common TRAEs included thyroid dysfunction, rash, and bone marrow suppression. The safety profile appeared manageable and consistent with known irAEs [93]. These early data are encouraging, especially considering the promising activity previously reported with cadonilimab in cervical cancer, suggesting potential for broader application in gynecologic malignancies [109].

Tebotelimab (MGD013) is a bispecific DART molecule that simultaneously targets PD-1 and LAG-3, two inhibitory immune checkpoints involved in T-cell exhaustion and immune evasion. It has been evaluated both as monotherapy and in combination with margetuximab, an Fc-engineered anti-HER2 mAb. The rationale for combining these two agents arises from preclinical evidence showing that margetuximab not only enhances ADCC compared to trastuzumab, through increased affinity for the activating FcγRIIIA (CD16A) and decreased affinity for the inhibitory FcγRIIIB (CD32B), but also leads to the upregulation of PD-L1 and LAG-3 on immune effector cells. This immunomodulatory effect could potentially dampen the anti-tumor immune response unless counterbalanced by immune checkpoint blockade. Therefore, the addition of tebotelimab was hypothesized to simultaneously block both PD-1/PD-L1 and LAG-3 inhibitory axes, thereby restoring T-cell effector function and amplifying HER2-directed ADCC. In clinical trials, the combination demonstrated promising activity in patients with HER2-positive tumors, many of whom were heavily pretreated with prior HER2-targeted therapies. The confirmed ORR was 19% (14/72; 95% CI: 11–30), with responses observed irrespective of baseline PD-L1 expression. Among patients without prior anti-HER2 therapy (*n* = 21), the ORR was 33% (95% CI: 15–57), including responses in both PD-L1-positive and -negative tumors. In contrast, the ORR was 14% (95% CI: 6–27) among those who had received previous anti-HER2 treatment (*n* = 50). Notably, 18% of PD-L1-negative patients in this subgroup also responded, highlighting the potential for the combination to overcome resistance mechanisms even in immunologically “cold” tumors. Furthermore, among patients with no prior checkpoint inhibitor therapy, the ORR was 22%, whereas only 8% of patients with prior checkpoint blockade exposure responded, suggesting that the combination may be more effective in immunotherapy-naive settings. Interestingly, most confirmed responses occurred in PD-L1-negative tumors, with 24% ORR in PD-L1-negative and 33% in PD-L1-positive patients, and 77% of responses in patients with known PD-L1 status were observed in PD-L1-negative tumors, underscoring the possibility that PD-L1 expression alone may not be a reliable predictor of response in this context. Tebotelimab monotherapy yielded more modest results in the ovarian cancer cohort, with an ORR of 11% (4/36; 95% CI: 3–26). To investigate potential biomarkers of response, exploratory immunohistochemical analyses were performed to assess PD-L1 and LAG-3 expression; however, no statistically significant association with clinical response was observed. In contrast, gene expression profiling using 32 predefined NanoString IO 360 immune-related signatures revealed a stronger correlation between clinical benefit and an IFN-γ-regulated immune proteasome signature, suggesting that a pre-existing inflamed TME may be critical for response. Importantly, tebotelimab demonstrated an acceptable safety profile, comparable to anti-PD-1 monotherapy, contrasting with the higher rates of irAEs observed with dual ICI combinations. Combining tebotelimab with margetuximab, which engages NK cells and promotes IFN-γ-driven LAG-3 upregulation, may further enhance ADCC and overcome resistance mechanisms. However, the trial is based on limited sample sizes and lacks a therapeutic control arm, underscoring the need for larger, controlled clinical trials to validate this strategy and identify predictive biomarkers such as baseline LAG-3 expression or IFN-γ-regulated gene signatures [94].

In patients with platinum-sensitive, recurrent ovarian cancer who are *gBRCA1/2* wild-type, the combination of PARPis with ivonescimab (AK112), bsAbs targeting both PD-1 and VEGF, has been investigated. This strategy aims to optimize anti-tumor effects by simultaneously addressing immune suppression and angiogenesis. Promising results have been reported in non-small cell lung cancer, where AK112 outperformed pembrolizumab as frontline therapy for PD-L1-positive advanced disease, and it also appears to have effects in endometrial cancer. Preliminary activity of ivonescimab has been reported in a phase I trial involving 19 patients with platinum-resistant ovarian cancer, with 5 patients (26.3%) showing a PR. The combination of PD-1/PD-L1 and VEGF inhibition has already been validated in multiple tumor types, supporting the rationale for this dual blockade. The safety profile of ivonescimab appears manageable, with grade 3 hypertension being the most frequently reported high-grade AEs, consistent with the known toxicities of VEGF-targeted therapies. However, given the limited sample size and the early-phase nature of the study, further research is needed to determine whether these response rates will be confirmed in larger, more robust trials [95, 110, 111]. The use of bsAbs with ICIs mechanism as monotherapy or in combination with chemotherapy or other immunotherapy agents for the treatment of ovarian cancer and other malignancies is being evaluated in an ongoing trial (NCT06522828). Two other trials have been completed, and we are currently awaiting the publication of their results (NCT05788484, NCT03849469).

Combinations of bsAbs with ADCs are being actively investigated. One such example is AZD8205, an ADC targeting B7-H4, currently in a phase I/IIa trial for advanced or metastatic solid tumors. AZD8205 consists of a human anti-B7-H4 antibody conjugated to a topoisomerase I inhibitor (TOPIi) payload. B7-H4, a transmembrane protein that suppresses T-cell activity, is overexpressed in cancers such as breast, ovarian, endometrial, and cholangiocarcinoma, and is associated with poor prognosis. AZD8205 is being evaluated both as monotherapy and in combination with AZD2936, a TIGIT/PD-1 bsAbs, particularly in ovarian and endometrial cancers (NCT05123482).

### Endometrial and cervical cancer

Unlike ovarian cancer, there are currently few ongoing studies evaluating bsAbs in endometrial and cervical cancers (Table 2).

**Table 2:** Summary of main clinical trials investigating bsAbs in cervical and ECs.

Drug name	Targets	Setting	Phase	*n*	Results	TEAEs/TRAEs/irAEs G ≥ 3* (in at least 5% of pts) and any grade CRS/IRR/ICANS** (%)
TAA mechanism						
Zanidatamab (ZW25) [54]	HER2 (ECD2) × HER2 (ECD4)	R/M EC/carcinosarcoma ≤ 2 lines	2	16	ORR: 6.2% mPFS: 1.7 mths (90% CI: 1.6–4.1 mths) OS: 14.5 mths (90% CI: 11.2–not estimable)	TRAEs G ≥ 3 no events ≥ 5% Any grade IRR 18.8% CRS/ICANS NR
ICI mechanism						
Cadonilimab (AK104)/placebo + cht +/– bev COMPASSION-16 [109, 112]	PD-1 × CTLA-4	R/M CC 1 line	3	445 (all pts) 222 (cadonilimab group) 223 (placebo group)	ORR: 79% vs. 68% mPFS: 13.3 vs. 8.2 mths (HR 0.62; 95% CI: 0.49–0.79, *p* < 0.0001) mOS: NA vs. 22.8 mths (HR 0.64; 95% CI: 0.48–0.86, *p* = 0.0011)	TRAEs G ≥ 3 cadonilimab vs. placebo a. neutropenia 41% vs. 46% b. anemia 15.9% vs. 24.2% c. platelet count decreased 14% vs. 12% d. diarrhea 2% vs. 1% e. hypokalemia 5.8% vs. 4.1% f. hypertriglyceridemia 6.2% vs. 4.1% g. hypertension 6.6% vs. 9.6% irAEs G ≥ 3 cadonilimab vs. placebo a. no events ≥ 5% Any grade IRR 11.9% vs. 2.3% CRS/ICANS NR
Ivonescimab (AK112/SMT112) [95]	PD-1 × VEGF	R EC	1	51 (all pts) 3 (EC)	ORR: 4.26%	TRAEs G ≥ 3 hypertension 13.7% ALT increased 5.2% irAEs G ≥ 3 no events ≥ 5% Any grade IRR 7.8% CRS/ICANS NR
Tebotelimab (MGD013) +/– margetuximab [94]	PD-1 × LAG-3	R/M CC 1 line	1	277 (all pts) Monotherapy: 17 (EC) Combination: 1 (EC)	ORR: 0	Monotherapy TRAEs G ≥ 3 a. no events ≥ 5% irAEs G ≥ 3 a. no events ≥ 5% any grade a. IRR 7.4% b. CRS/ICANS NR Combination TRAE G ≥ 3 a. AST increased 5% irAEs G ≥ 3 a. no events ≥ 5% any grade a. IRR 7.1% b. CRS/ICANS NR

The Table provides a comprehensive overview of the main clinical trials evaluating bispecific antibodies (bsAbs) in cervical and ECs. Only studies with available clinical data in these tumor types are reported, with a focus on target specificity, study phase, treatment combinations, and preliminary efficacy or safety outcomes, when available. *: Some trials reported treatment-related adverse events (TRAEs), other treatment-emergent adverse events (TEAEs), and immune-related adverse events (irAEs). Only grades ≥ 3 observed in more than 5% of patients are included in this Table; **: adverse events of special interest (AESIs), including cytokine release syndrome (CRS), infusion-related reaction (IRR), and immune effector cell-associated neurotoxicity syndrome (ICANS), are listed in the Table regardless of grade and even if occurring in less than 5% of patients. G: grade; TAA: tumor-associated antigen; HER2: human epidermal growth factor receptor 2; ECD2: extracellular domain 2; ECs: endometrial cancers; ORR: objective response rate; mPFS: median progression-free survival; mths: months; CI: confidence interval; OS: overall survival; mOS: median OS; NR: not reported; ICI: immune checkpoint inhibitor; cht: chemotherapy; bev: bevacizumab; PD-1: programmed cell death protein 1; CTLA-4: cytotoxic T-lymphocyte-associated protein 4; R/M: recurrent/metastatic; CC: cervical cancer; pts: patients; HR: hazard ratio; NA: not available; VEGF: vascular endothelial growth factor; R: recurrent; ALT: alanine aminotransferase; LAG-3: lymphocyte activation gene 3; AST: aspartate aminotransferase.

#### TAA mechanism

HER2 overexpression in endometrial cancer has been associated with worse OS, highlighting the need for effective targeted therapies in this subset of patients. In advanced HER2-positive endometrial carcinoma, the addition of trastuzumab, a mAb targeting the extracellular domain of HER2, to standard chemotherapy with carboplatin and paclitaxel has demonstrated clinical benefit [113]. Despite this progress, treatment options beyond first-line trastuzumab-based regimens remain limited, particularly for patients with disease progression following platinum-based chemotherapy. Recently, the ADC T-DXd has shown promising efficacy, and confirmatory trials are ongoing. Among emerging therapeutic approaches, zanidatamab (ZW25), a bsAb that binds two non-overlapping epitopes of HER2, has been explored in HER2-positive endometrial cancer. In a phase II study enrolling 16 patients with HER2-overexpressing metastatic endometrial carcinoma or carcinosarcoma, the ORR was modest (6.2%), with only one PR reported. However, the clinical benefit rate at 24 weeks was 37.5%. Due to limited efficacy, the study did not progress to the second stage. Importantly, the only responding patient had not received prior trastuzumab, which may suggest potential benefit in trastuzumab-naive settings. The safety profile of zanidatamab was favorable, with most AEs being grade 1–2; no grade ≥ 3 toxicities were reported. The most common were diarrhea, nausea, and infusion-related reaction (IRR). However, the study presented several limitations. Firstly, repeat HER2 testing before treatment initiation was not consistently performed, raising concerns about potential HER2 heterogeneity or loss of expression over time. Secondly, unlike ADCs, zanidatamab lacks a cytotoxic payload, which may partially explain the limited anti-tumor activity observed in this study [54]. To address these challenges, the bsAb is currently being evaluated in combination with chemotherapy in other HER2-expressing tumors. In a phase II, open-label trial, zanidatamab combined with the physician’s choice of chemotherapy showed promising activity in HER2-positive gastroesophageal adenocarcinoma [114]. Based on these results, a randomized phase III trial (HERIZON-GEA-01) is evaluating zanidatamab in combination with chemotherapy, with or without tislelizumab (humanized IgG4 mAb directed against PD-1 protein), against the current standard of care (trastuzumab plus chemotherapy). The results of these trials might open opportunities for other tumor types, including gynecological cancers [115].

#### ICE mechanism

In addition to the bsAbs already discussed, other promising candidates in endometrial cancer include those targeting CLDN6. CLDN6 is an oncofetal tight junction protein typically silenced in adult tissues but aberrantly re-expressed in a subset of endometrioid endometrial cancer, where it is associated with aggressive pathological features such as deep myometrial invasion, lymphovascular space involvement, and advanced-stage disease. High CLDN6 expression correlates with poor prognosis and may drive tumor proliferation and migration, making it an attractive therapeutic target for antibody-based strategies [116]. Preclinical studies support the oncogenic role of CLDN6. In vitro silencing of CLDN6 in HEC-1-B endometrial cancer cells significantly reduced proliferation, migration, and invasion, accompanied by downregulation of the PI3K/AKT/mTOR pathway. These findings are consistent with prior evidence linking CLDN6 to increased tumor aggressiveness in various solid tumors, including gastric, esophageal, and lung cancers. While the exact molecular mechanisms remain to be fully elucidated, CLDN6 may influence tumor progression by disrupting tight junction integrity, altering ionic homeostasis at the lateral membrane, and modulating the EMT [117]. Based on this rationale, bsAbs targeting CLDN6 are now in clinical development. XmAb541, designed to engage CD3 on T-cells and CLDN6 on tumor cells, is being evaluated in gynecologic malignancies such as ovarian and uterine cancers (NCT06276491). Similarly, CTIM-76 is undergoing phase I/II investigation in patients with platinum-resistant ovarian cancer and other CLDN6-positive advanced solid tumors, including endometrial cancer (NCT06515613). Preliminary data from this phase I trial have been presented but not yet formally published. Among TEAEs, CRS was reported in approximately 25% of patients, while no other significant safety concerns were noted. B7-H4 also known as DD-O110, B7x, B7 superfamily member 1 (B7S1), or V-set domain containing T-cell activation inhibitor 1 (VTCN1), is a type I transmembrane glycoprotein and a member of the B7 family of immune checkpoint molecules [118]. Under physiological conditions, its expression is largely restricted to antigen-presenting cells, where it acts as a negative regulator of T-cell-mediated immunity [119]. In the context of malignancy, B7-H4 is frequently overexpressed in gynecologic tumors, including up to 71.5% of endometrial cancers, particularly within p53-abnormal and NSMP subtypes [120]. Notably, its expression is generally low or absent in normal tissues, making it an appealing therapeutic target. Functionally, B7-H4 contributes to an immunosuppressive TME by inhibiting T-cell activation, promoting regulatory T-cell (Treg) expansion, and recruiting TAMs, thereby facilitating immune evasion and tumor progression [121]. However, the prognostic significance of B7-H4 in endometrial cancer remains controversial. Zong et al. [120] reported that B7-H4 positivity was associated with improved relapse-free survival and disease-specific survival (DSS), identifying it as an independent prognostic factor. Conversely, data from Gorzelnik et al. [122] suggested that high B7-H4 expression was associated with poorer OS compared to B7-H4-low patients. Moreover, VTCN1 hypomethylation and upregulation have been linked to less favorable outcomes [122]. In cervical cancer, B7-H4 is rarely expressed in normal epithelium but is upregulated in over 80% of malignant lesions, especially during progression from cervical intraepithelial neoplasia (CIN) III to invasive carcinoma [121]. Its expression correlates with reduced CD8+ T-cell infiltration, increased Treg presence, and elevated serum levels of soluble B7-H4 (sB7-H4), highlighting its role as a mediator of immune suppression [120]. Some studies associate B7-H4 expression with a worse prognosis, while others report conflicting findings. Notably, B7-H4 may also contribute to HPV-driven oncogenesis. Experimental silencing of VTCN1 in cervical cancer models resulted in increased tumor suppressor retinoblastoma protein mRNA levels and decreased expression of the HPV E7 oncoprotein, implicating B7-H4 in the E7/Rb regulatory axis [119]. Altogether, these data support B7-H4 and CLDN6 as compelling immunotherapeutic targets in endometrial and cervical cancer. Ongoing trials with bsAbs against these TAAs are expected to provide insights into their clinical utility, particularly in tumors resistant to conventional ICIs or standard therapies.

#### ICI mechanism

Cadonilimab is a bsAb targeting PD-1 and CTLA-4, designed to enhance anti-tumor immune responses by simultaneously blocking these immune checkpoints. The phase III COMPASSION-16 trial evaluated the efficacy of cadonilimab, a bsAb targeting PD-1 and CTLA-4, in combination with platinum-based chemotherapy with or without bevacizumab, in patients with persistent, recurrent, or metastatic cervical cancer. In this randomized, double-blind trial, 445 patients with no prior systemic treatment for cervical cancer were assigned to receive either cadonilimab (10 mg/kg every 3 weeks) or placebo, both in combination with chemotherapy (platinum ± paclitaxel), with optional addition of bevacizumab (15 mg/kg). Subgroup analyses were performed to assess treatment benefit based on PD-L1 expression levels, categorized by combined positive score (CPS; < 1, ≥ 1, and ≥ 10). At a median follow-up of 26 months, cadonilimab demonstrated consistent improvement in both PFS and OS across all predefined subgroups. These findings suggest that the addition of cadonilimab provides clinical benefit regardless of patients’ age, prior treatment history, PD-L1 expression level, or chemotherapy regimen [112]. Regarding safety, the most frequently reported AEs of any grade and grade ≥ 3 in both treatment groups were decreased neutrophil count, decreased white blood cell count, and anemia. AE led to treatment discontinuation in 63 patients (28%) in the cadonilimab group and 23 patients (11%) in the placebo group. Death due to AEs occurred in 12 patients (5%) in the cadonilimab group, of which 9 (4%) were considered treatment-related, and in 7 patients (3%) in the placebo group, of which 6 (3%) were treatment-related. TRAEs were observed in 225 patients (> 99%) receiving cadonilimab and in all 219 patients (100%) in the placebo group, with grade ≥ 3 treatment-related AEs occurring in 186 patients (82%) and 173 patients (79%), respectively. Notably, four participants originally randomized to the placebo group inadvertently received a single dose of cadonilimab and were subsequently analyzed within the cadonilimab group for safety assessments. irAEs occurred more frequently in the cadonilimab group (46%) compared to placebo (7%), with grade ≥ 3 irAEs in 10% and < 1% of patients, respectively. No grade 5 events were reported in either arm. These data reflect the previously reported safety profile; updated safety outcomes have not yet been made available alongside the most recent efficacy results [109].

Ivonescimab is an anti PD-1/VEGF antibody that combines immune checkpoint blockade with angiogenesis inhibition. By targeting PD-1, it restores T-cell activation and enhances anti-tumor immune responses, while inhibiting VEGF reduces tumor vascularization and improves immune cell infiltration. Ivonescimab has demonstrated promising efficacy in dMMR and pMMR endometrial cancers, as well as in pMMR colorectal cancer and non-small cell lung cancer. In a small cohort of three patients with endometrial cancer, one patient with an dMMR tumor achieved a PR, another with a pMMR tumor also achieved a durable PR, and the third patient with unknown MMR status achieved stable disease for 16 weeks, with an 8.5% reduction in tumor burden. Although the sample size is limited, these findings suggest that the anti-tumor activity of ivonescimab in endometrial cancer may not be strictly dependent on MMR status. Similarly, the study involving MSS/pMMR colorectal cancer, ivonescimab monotherapy achieved an ORR of 11.1% among nine patients, a noteworthy result given the typical resistance of MSS colon rectal cancer to ICIs. The safety profile of ivonescimab was consistent with that observed for other anti-PD-1 and anti-VEGF agents, with no new safety signals reported [95]. Furthermore, two bsAbs targeting PD-1 and CTLA-4 are currently under investigation in endometrial cancer, either as monotherapy or in combination with chemo-radiotherapy (NCT06532539, NCT06522828). The DUET-4 study is evaluating XmAb22841, which simultaneously targets immune checkpoint receptors CTLA-4 and LAG-3 either alone or in combination with pembrolizumab across various solid tumors, including endometrial and cervical cancer (NCT03849469). Although the study has been completed, results have not yet been published and are currently awaited.

Tebotelimab (MGD013) is another one designed to simultaneously block PD-1 and LAG-3, aiming to enhance immune activation by overcoming immune exhaustion. It was evaluated in combination with margetuximab, an anti-HER2 antibody, in patients with platinum-resistant ovarian and cervical cancer; however, preliminary data did not show a confirmed ORR in these tumor types [94].

Additional studies are also investigating AK104 in other settings, such as in combination with radiotherapy for locally advanced disease and with the multi-target tyrosine kinase inhibitor anlotinib for pre-treated metastatic disease (NCT05687851, NCT05817214). The therapeutic landscape of cervical cancer is evolving rapidly, particularly with the integration of immunotherapy and molecularly targeted agents. Despite these advances, a significant proportion of patients with advanced or recurrent disease remain unresponsive to immune checkpoint blockade, especially PD-1/PD-L1 inhibitors [26, 27]. This underscores the need to identify and target alternative immunosuppressive mechanisms within the TME. One of the most critical mediators in this context is TGF-β, a pleiotropic cytokine with dual roles in both normal physiology and tumorigenesis. TGF-β regulates key cellular processes, including proliferation, apoptosis, angiogenesis, and tissue repair. While it exerts tumor-suppressive functions during early neoplastic transformation, in later stages it facilitates tumor progression by promoting EMT, immune evasion, and re-modeling of the TME [123]. Notably, TGF-β contributes to the recruitment and activation of immunosuppressive cell populations such as Tregs, TAMs, and myeloid-derived suppressor cells, thereby limiting anti-tumor immune responses [123, 124]. In HPV-driven cancers such as cervical cancer, the oncogenic proteins E6 and E7 can enhance TGF-β signaling, fostering an immunosuppressive niche that supports tumor growth [125]. Additionally, TGF-β indirectly upregulates PD-L1 expression via non-SMAD pathways, including PI3K/Akt, MAPK/ERK, and JAK/STAT signaling. Under hypoxic conditions, TGF-β further interacts with hypoxia-inducible factor 1-alpha (HIF-1α), reinforcing PD-L1 expression and contributing to immune resistance. Despite promising preclinical results, monotherapies targeting the TGF-β pathway, whether via receptor kinase inhibitors or anti-TGF-β mAb, have shown limited clinical success, with responses restricted to small patient subsets. This has led to the development of bsAb that simultaneously targets both PD-L1 and TGF-β, aiming to overcome resistance through dual pathway blockade [123]. YM101 is a bsAb that combines an anti-PD-L1 scFv with a TGF-β receptor II extracellular domain. In preclinical studies, YM101 demonstrated potent anti-tumor activity by reversing TGF-β-induced immunosuppression and enhancing T-cell-mediated cytotoxicity. Notably, YM101 promoted the formation of an inflamed TME by increasing the numbers of TILs and dendritic cells, elevating the M1/M2 macrophage ratio, and enhancing cytokine production in T-cells [126]. BiTP is structurally like YM101, designed to target both human PD-L1 and TGF-β. In murine models of triple-negative breast cancer, BiTP effectively reduced peritumoral collagen deposition and promoted T-cell infiltration, leading to significant anti-tumor effects [127]. Encouraged by these preclinical results, different trials are underway to evaluate the safety and efficacy of these agents in patients with advanced solid tumors, including cervical cancer (NCT05028556; NCT05537051). These bsAbs offer a rational strategy to remodel the TME, overcome immune resistance, and improve clinical outcomes for patients with cervical cancer. Their ability to simultaneously target PD-L1 and TGF-β pathways addresses two critical mechanisms of immune evasion, potentially enhancing the efficacy of existing immunotherapies and providing a novel therapeutic approach for this challenging malignancy.

## The safety profile associated with bsAbs in gynecologic malignancies

Despite bsAbs have emerged as promising immunotherapeutic agents in the treatment of gynecologic cancers, their mechanism of action is associated with some unique and sometimes severe toxicities, requiring careful analysis and management strategies to optimize their clinical use [128]. These AEs, mainly related to T-cell-engaging bsAbs, can affect the skin (rash, itching), gastrointestinal tract (colitis), liver (hepatitis), endocrine glands (thyroiditis, adrenal insufficiency), and lungs (pneumonitis) [129]. Additionally, blood-related toxicities like anemia, neutropenia, and thrombocytopenia are common, along with fatigue, which is reported in nearly all clinical trials involving bsAbs [130]. Notably, due to their rarity and stronger connection to bsAbs, CRS, IRRs, and ICANS are of particular concern (Tables 1 and 2). CRS is the most common and serious toxicity, especially in bsAbs that target CD3 on T-cells. It involves a systemic inflammatory response caused by rapid T-cell activation, macrophage stimulation, and cytokine production, mainly IFN-γ, IL-6, TNF-α, and IL-10. These cytokines lead to vascular leakage, fever, low blood pressure, and, in severe cases, multi-organ failure. IFN-γ contributes to fever, chills, and fatigue; IL-6 is involved in hypotension and capillary leak; TNF-α is linked to gastrointestinal symptoms and lung injury. Typically, CRS occurs within 48 h after the first dose of bsAb and tends to lessen in later cycles [131]. In the reviewed studies, CRS was mostly reported as grade 1 or 2, with no cases of grade 3 documented [85–87, 90, 91]. Managing CRS requires close monitoring, prompt supportive care, and often anti-inflammatory drugs. Tocilizumab, an IL-6 receptor blocker, is approved to treat CRS. While preventive use of tocilizumab isn’t currently recommended, it is being studied. Corticosteroids like dexamethasone are suggested for CRS prevention strategies [132]. When CRS occurs, dose adjustments are usually made. Notably, tocilizumab was only reported in the study involving JNJ-78306358 [90]. IRRs have also been observed, generally limited to grade 1 or 2 events and only one case of grade 3 with ABBV-428 [54, 87, 89, 90, 94, 95]. They typically happen within 10 min to 4 h of infusion, but can be delayed up to 24 h. Symptoms include chills, difficulty breathing, flushing, nausea, chest discomfort, and vomiting. These reactions are usually self-limiting and manageable with supportive care, such as antihistamines, corticosteroids, pain relievers, oxygen, and H2 antagonists. Prevention strategies involve dividing the initial dose over several days, slowing infusion rates, and premedicating with corticosteroids [133]. Specific management details are not consistently provided across studies. Although ICANS is a recognized AE in T-cell redirecting therapies, only one case exceeding grade 3 severity was reported in the reviewed bsAb trials, specifically with ubamatamab [85]. ICANS is thought to result from cytokine-driven endothelial activation, disruption of the blood-brain barrier, and subsequent neurotoxicity. Early symptoms can be subtle and include tremors, mild speech difficulty, motor issues, writing problems, and lethargy, with speech problems often indicating severe neurotoxicity. Symptoms can rapidly worsen to delirium, seizures, or coma within hours or days, while the long-term effects are still unclear. Treatment focuses on close neurological monitoring and supportive care; severe cases may require intensive care and airway management [133]. Unlike CRS, which responds to tocilizumab, ICANS does not respond well because it poorly penetrates the blood-brain barrier. Corticosteroids are the primary treatment, with tocilizumab reserved for cases with concurrent CRS. The ubamatamab study did not specify how to manage the severe ICANS case [85]. Several bsAbs have been linked to irAEs requiring intervention. For example, tebotelimab was associated with immune-mediated hepatitis and increased lipase levels; cadonilimab and vudalimab required dose reductions due to irAEs; and ivonescimab was linked to myocarditis, managed by dose reduction without stopping treatment [92, 94, 95, 112]. Another safety concern is on-target, off-tumor toxicity, which happens when bsAbs target TAAs also present on healthy tissues. This can cause unintended immune-mediated tissue damage and depends on antigen choice, expression levels, and bsAb binding strength. To enhance selectivity and reduce toxicity, strategies include antigen density discrimination, dual-antigen targeting, affinity tuning, and switchable bsAb formats activated in the TME [134]. These findings highlight the importance of thorough antigen validation and developing next-generation bsAbs with better tumor targeting and fewer off-tumor effects. So far, no consistent links between biomarkers and toxicity have been found in early-phase gynecologic cancer trials. As these therapies advance, discovering predictive biomarkers for both effectiveness and side effects will be vital for personalized treatment. Although CRS and other irAEs are acknowledged in trials, detailed prevention and mitigation strategies are often missing. Future efforts should focus on optimizing dosing schedules and early intervention methods. For the safe and effective use of bsAbs in gynecologic cancers, careful toxicity monitoring, dose management, and prompt AEs treatment are essential. Ongoing research should aim to improve antigen targeting, identify predictive biomarkers, and develop early intervention strategies. Continuous clinical studies will be crucial to enhance safety and maximize the therapeutic benefits of bsAbs in gynecologic oncology.

## Future directions

The clinical development of bsAbs has marked a significant progress in the therapeutic landscape of gynecologic malignancies. Due to their innovative mechanisms of action and promising efficacy, substantial efforts have been dedicated to understanding potential mechanisms of resistance that may compromise clinical outcomes. Simultaneously, new emerging strategies are being investigated to overcome these limitations, including the design of next-generation bsAb formats and rational combination regimens [133]. One of the most prominent resistance mechanisms involves the loss or downregulation of target antigen expression, which reduces bsAb engagement with tumor cells and may result from tumor evolution under therapeutic pressure [135, 136]. Another critical mechanism is the loss of MHC class I molecules, which impairs antigen presentation and T-cell recognition, particularly in tumors targeted by bsAbs against immunogenic antigens such as HER2 [137, 138]. Moreover, immune evasion can occur through the upregulation of immune checkpoint proteins (e.g., PD-L1, CTLA-4) and TME, which suppress T-cell activation despite bsAb-mediated redirection [139]. As large and complex protein therapeutics, bsAbs are also susceptible to the development of anti-drug antibodies (ADAs). These humoral responses may generate neutralizing antibodies (NAbs) that block bsAb binding domains, or non-NAbs that alter pharmacokinetics, trigger hypersensitivity reactions, or mediate complement-dependent toxicities. Although the incidence of ADA formation in bsAb therapy has not been systematically characterized, it remains a potential barrier to sustained efficacy [140]. The TME represents an additional and frequently underestimated mechanism of resistance to bsAbs. The TME is characterized by elevated interstitial fluid pressure, dense ECM deposition, and aberrant vasculature, all of which restrict the infiltration and diffusion of therapeutic agents. Although bsAbs are smaller than full-length IgG antibodies, they remain subject to barriers related to molecular size and limited target-site accessibility. A well-described phenomenon, the “binding-site barrier”, occurs when bsAbs are sequestered by peripheral tumor cells expressing the target antigen, preventing their deeper penetration into the tumor core. Furthermore, normal tissues with low-level antigen expression can act as “antigen sinks”, decreasing effective bsAb concentrations at the tumor site [134]. This mechanism is particularly relevant in gynecologic malignancies, which are often characterized by a dense, immunosuppressive, and heterogeneous TME [141]. To address these resistance mechanisms, several innovative strategies are under development. Combination therapies involving bsAbs, ICIs, ADCs, or adoptive cell therapies such as CAR-T and CAR-NK cells may promote synergistic anti-tumor activity (NCT02879695, NCT03340766, NCT02650713) [142–144]. An emerging strategy to overcome immune resistance in solid tumors, including gynecological cancers, involves developing trispecific antibodies that not only redirect T-cells to tumor cells but also deliver co-stimulatory signals and enhance pharmacokinetics [145]. One such example is NM21-1480 (also known as CS2006). This trispecific antibody simultaneously targets PD-L1, 4-1BB (CD137), and human serum albumin (HSA). By blocking PD-L1, NM21-1480 decreases immune suppression within the TME. At the same time, engaging 4-1BB provides a co-stimulatory signal that boosts T-cell activation and proliferation. The HSA-binding domain is designed to improve serum half-life and tissue distribution. This multi-modal approach aims to promote stronger anti-tumor immunity than traditional bispecifics. Another promising innovation involves protease-activated bsAb prodrugs, designed to remain inert in systemic circulation and become activated only in the TME by tumor-associated proteases such as matrix metalloproteinase 2 or matriptase. Compounds like folate receptor 1-T-cell bsAb (FOLR1-TCB) and protease-activated anti-FOLR1-TCB (Prot-FOLR1-TCB) have shown potent preclinical activity in ovarian cancer models, with reduced systemic toxicity [146]. Local bsAb delivery via mRNA lipid nanoparticles (LNPs) or oncolytic viruses offers a strategy to achieve sustained intratumoral expression while minimizing off-target effects. For instance, LNP-encoded BiTEs targeting B7-H3 × CD3 have demonstrated durable anti-tumor responses in preclinical models [147]. Another emerging concept involves using bsAbs to disrupt cancer cell proliferation by promoting the targeted degradation of key surface proteins. This idea is inspired by the development of bispecific or multispecific small-molecule proteolysis-targeting chimeras (PROTACs). In this context, bsAbs are engineered to bind both a surface protein involved in tumor growth or survival and a secondary molecule that facilitates internalization and subsequent degradation of the target, such as membrane-associated E3 ubiquitin ligases or the transferrin receptor. Through this dual engagement, bsAbs can induce the internalization of the target protein and direct it toward lysosomal or proteasomal degradation. While these antibody-PROTAC hybrids remain in early development stages and have not yet entered clinical trials, their modular design is fully compatible with current bsAb platforms [148, 149]. In parallel, bsAbs designed as synthetic cytokine agonists or incorporating split cytokine modules (e.g., IL-2, IL-15, or type I interferons) may offer potent, localized immune stimulation while reducing systemic toxicity, promoting T-cell proliferation, persistence, and memory formation [150]. As bsAb platforms continue to evolve, identifying predictive biomarkers, such as target antigen density, immune gene signatures, or TME composition, will be critical for optimizing patient selection and therapeutic success. Furthermore, determining the optimal sequence of bsAbs with other agents (e.g., ADCs, ICIs, or chemotherapy) remains undefined and warrants further investigation in future clinical trials [151]. While considerable progress has been made in common gynecologic malignancies such as endometrial, cervical, and epithelial ovarian cancers, rare subtypes, including vulvar, vaginal, and non-epithelial ovarian tumors, remain largely underexplored. Expanding bsAb research into these rarer entities, which often lack effective standard treatments, could address a major unmet clinical need and broaden the impact of this promising class of therapeutics.

## Conclusions

BsAbs represent a promising and innovative class of therapy with the potential to revolutionize the treatment landscape of many solid tumors, including gynecological cancers. By simultaneously targeting two distinct antigens, they uniquely redirect immune effector cells toward tumor cells while reshaping the immunosuppressive TME. This dual mechanism of action allows them to surpass the therapeutic potential of mAbs and ICIs alone. Importantly, bsAbs may act synergistically with other therapeutic strategies, including chemotherapy, immunotherapy, and radiotherapy. These combinations have the potential to intensify anti-tumor responses and overcome tumor heterogeneity and acquired resistance. Moreover, integrating bsAbs with emerging modalities such as ADCs and CAR-T cell therapies holds considerable promise. However, the optimal sequencing and combination strategies remain largely undefined and warrant further clinical investigation. Despite these opportunities, bsAbs are not without challenges. Indeed, their unique toxicity profiles can limit their broader clinical application. A comprehensive understanding of these AEs and their underlying biological mechanisms is essential to guide the development of effective prophylactic and therapeutic interventions.

Future research should focus on refining combination strategies, enhancing tumor specificity, minimizing AEs, and identifying reliable predictive biomarkers. Although bsAbs represent a promising therapeutic modality in gynecologic oncology, their successful integration into clinical practice will require continued translational research and rigorous clinical validation.

## Abbreviations

ADAs: anti-drug antibodies
ADCC: antibody-dependent cell-mediated cytotoxicity
ADCP: antibody-dependent cellular phagocytosis
ADCs: antibody-drug conjugates
AEs: adverse events
BiTEs: bispecific T-cell engagers
*BRCA*: breast cancer susceptibility gene
bsAbs: bispecific antibodies
bsADCs: bispecific antibody drug conjugates
CA-125: cancer antigen 125
CAR-T: chimeric antigen receptor T-cell
Ch1: the first constant heavy domain
Cl: constant light domain
CLDN6: claudin-6
CrossMab: cross-over monoclonal antibody
CRS: cytokine release syndrome
ctDNA: circulating tumor DNA
CTLA-4: cytotoxic T-lymphocyte-associated protein 4
DART: dual affinity re-targeting
DCR: disease control rate
DLT: dose-limiting toxicity
dMMR: deficient mismatch repair
DoR: duration of response
DVD: dual variable domain
ECM: extracellular matrix
EMT: epithelial-to-mesenchymal transition
EpCAM: epithelial cell adhesion molecule
EVs: extracellular vesicles
Fab: fragment antigen-binding
Fc: fragment crystallizable
FOLR1-TCB: folate receptor 1-T-cell bispecific antibody
FRα: folate receptor alpha
HER2: human epidermal growth factor receptor 2
HLA-G: human leukocyte antigen-G
HPV: human papillomavirus
HR: hazard ratio
HRD: homologous recombination deficiency
HSA: human serum albumin
ICANS: immune effector cell-associated neurotoxicity syndrome
ICE: immune cell engager
ICIs: immune checkpoint inhibitors
ImmTAC: immune-mobilizing monoclonal T-cell receptor against cancer
irAEs: immune-related adverse events
IRR: infusion-related reaction
KiH: knobs-into-holes
LAG-3: lymphocyte activation gene 3
LNPs: lipid nanoparticles
mAbs: monoclonal antibodies
MHC: major histocompatibility complex
MSI-H: microsatellite instability-high
MUC16: mucin 16
NAbs: neutralizing antibodies
Nbs: nanobodies
NK: natural killer
NSMP: no specific molecular profile
ORR: objective response rate
OS: overall survival
PARPis: PARP inhibitors
PD-1: programmed cell death protein 1
PD-L1: programmed death-ligand 1
PFS: progression-free survival
pMMR: proficient mismatch repair
PR: partial response
PRAME: preferentially expressed antigen in melanoma
PROTACs: proteolysis-targeting chimeras
PuFS: puncture-free survival
scFv: single-chain variable fragment
sdAbs: single-domain antibodies
TAAs: tumor-associated antigens
TAMs: tumor-associated macrophages
TCF: tumor cell fraction
T-DXd: trastuzumab deruxtecan
TEAEs: treatment-emergent adverse events
TGF-β: transforming growth factor-beta
TIGIT: T-cell immunoreceptor with Ig and ITIM domains
TILs: tumor-infiltrating lymphocytes
TME: tumor microenvironment
TRAEs: treatment-related adverse events
Treg: regulatory T-cell
VEGF: vascular endothelial growth factor
Vh: variable regions of the heavy
Vl: variable regions of the light
VTCN1: V-set domain containing T-cell activation inhibitor 1

## Declarations

### Copyright

© The Author(s) 2025.
